# HGF-mediated crosstalk between cancer-associated fibroblasts and *MET*-unamplified gastric cancer cells activates coordinated tumorigenesis and metastasis

**DOI:** 10.1038/s41419-018-0922-1

**Published:** 2018-08-29

**Authors:** Xusheng Ding, Jun Ji, Jinling Jiang, Qu Cai, Chao Wang, Min Shi, Yingyan Yu, Zhenggang Zhu, Jun Zhang

**Affiliations:** 1 Department of Oncology, Ruijin Hospital, Shanghai Jiaotong University School of Medicine, No. 197 Ruijin Er Road, Shanghai, 200025 China; 2 Department of Surgery, Shanghai Institute of Digestive Surgery, Ruijin Hospital, Shanghai Jiaotong University School of Medicine, No. 197 Ruijin Er Road, Shanghai, 200025 China

## Abstract

Cancer-associated fibroblasts (CAFs) are important components of tumor stroma and play a key role in tumor progression. CAFs involve in crosstalk with tumor cells through various kinds of cytokines. In the present study, we screened hepatocyte growth factor (HGF) as a cytokine predominantly originating from CAFs. CAFs-derived HGF was found to promote *MET*-unamplified gastric cancer (GC) proliferation, migration, and invasion through the activation of HGF/c-Met/STAT3/twist1 pathway. It also activated interleukin (IL)-6/IL-6R/JAK2/STAT3/twist1 pathway by up-regulating IL-6R expression. As IL-6 was also found to upregulate c-Met expression, we identified the cooperation of HGF and IL-6 in enhancing the characteristics of CAFs. In vivo experiments revealed that CAFs-derived HGF promoted tumorigenesis and metastasis of *MET*-unamplified GC. Gene set enrichment analysis (GSEA) was performed to confirm our findings. Our study found that the increased expression of HGF in CAFs induced by *MET*-unamplified GC contributed to the malignant phenotype of both *MET*-unamplified GC and CAFs in tumor microenvironment.

## Introduction

The tumor stroma, which comprises the extracellular matrix (ECM) and various kinds of stromal cells, such as fibroblasts, inflammatory cells, and endothelial cells, has a marked influence on tumor initiation and progression^1,2^. Fibroblasts, the predominate cells in stroma, display their crucial roles in maintaining ECM and adjacent epithelia homeostasis through direct stromal-epithelial contact and secretion of cytokines^3^. However, following neoplastic transformation of epithelia, local normal fibroblasts (NFs) were educated to be CAFs, which were phenotypically and functionally distinguishable from their normal counterparts with the enhanced marker expressions, mainly alpha smooth muscle actin (α-SMA) and others including fibroblast activation protein (FAP), C-X-C motif chemokine ligand-12/ stromal cell-derived factor-1 (CXCL-12/SDF-1), fibroblast-specific protein-1 (FSP-1), platelet-derived growth factor receptor-α (PDGFRα), and platelet-derived growth factor receptor-β (PDGFRβ)^3,4^. After being transformed, the capacity of CAFs is enhanced in promoting malignant processes via secreting growth factors and inflammation factors^5,6^.

An increasing number of articles reported that the crosstalk between tumor and stromal cells created an appropriate microenvironment for tumor growth and metastasis^3,7^. CAFs actively communicated with cancer cells and promoted tumor progression through cytokines such as HGF, IL-6, TGF-β, VEGF, FGF, and CXCL12^8–10^. Among the cytokines secreted by CAFs, HGF and IL-6 participated in phenotype modulation of cancer cells in many solid tumors^11,12^. HGF was originally determined to be a stimulating factor that promoting the mitosis of hepatocytes, followed by discovering its effect on accelerating wound healing and histodifferentiation^13–15^. HGF from fibroblasts and c-Met from tumor cells formed a signaling pathway, which was intensely correlated with proliferation, metastasis, and angiogenesis^16,17^. Several studies reported that high-c-Met expression tumor cells due to copy number alteration of proto-oncogene *MET* showed no responses to the stimulation of their ligands^18,19^, while the opposites remained^8,20^. On the other hand, IL-6, a multifunctional cytokine, which was originally determined to be a regulator of immune and inflammatory responses^21^, was proved to be another important mediator linking epithelial cells and stromal cells^9,12^. IL-6 bound to a cell-surface type I cytokine-receptor complex consisting of IL-6Rα chain (IL-6-R) and a common cytokine-receptor signal-transducing subunit gp130, and then activates STAT3 with the phosphorylation of Tyr705 via the JAK2 signaling pathway^22,23^. It has been well elucidated that enhanced effect of IL-6/JAK2/STAT3 axis increased the chance of oncogenesis of ovarian, renal, and breast cancers^24–26^. In the present study, we identified the cooperation of HGF and IL-6 both on *MET*-unamplified GC and fibroblasts. Furthermore, we also characterized the molecular mechanisms underlying the cooperative effect on tumor growth and metastasis.

## Results

### HGF is predominantly derived from CAF in GC microenvironment

**Fig. 1:**
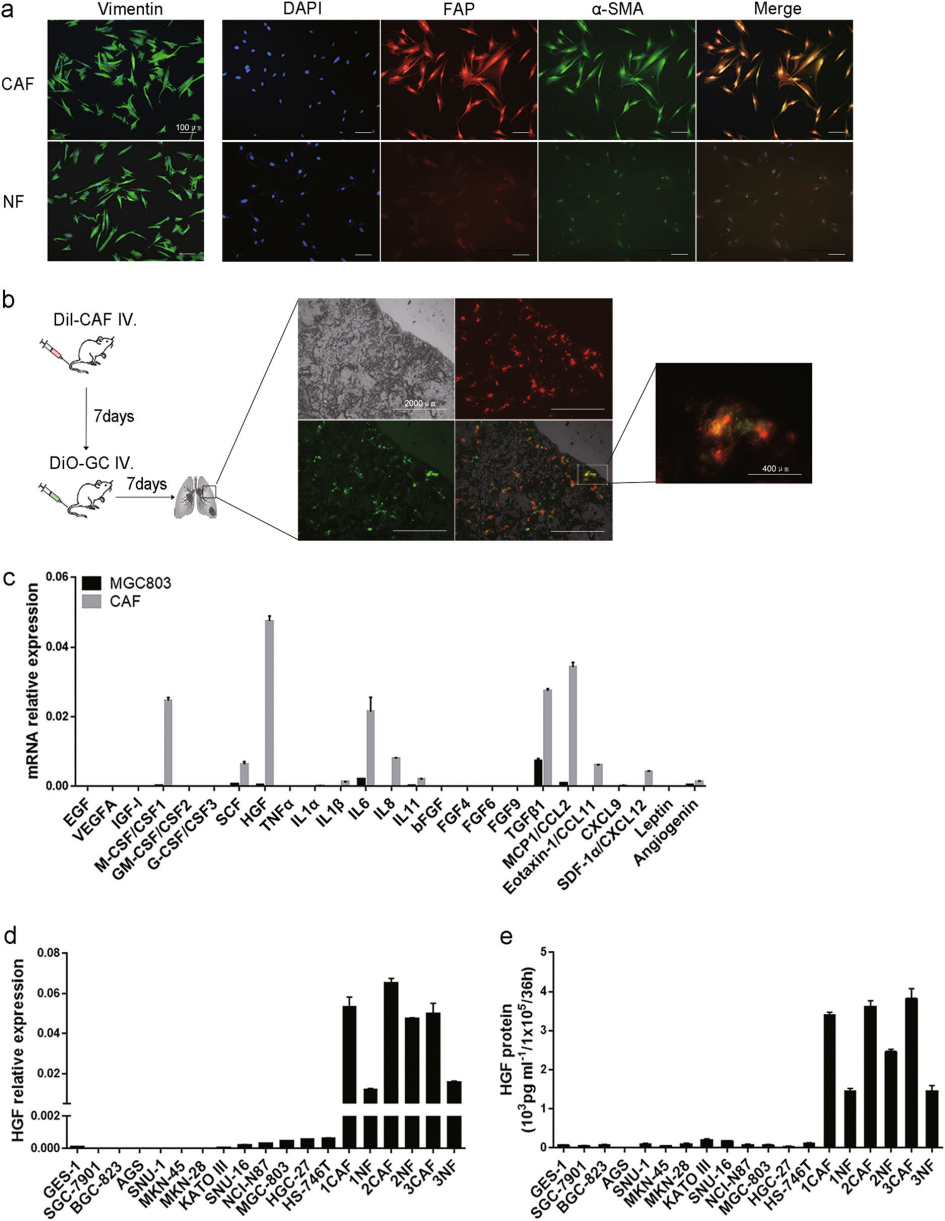
CAFs promote GC metastasis and are predominant resource of HGF expression. **a** Representative images of immunofluorescence staining of primary CAFs and NFs with Vimentin (green), DAPI (blue), FAP (red), and α-SMA (green). Scale bars, 100 μm. **b** Dil (red) labeled CAFs and DiO (green) labeled GC were subjected to nude mice and the images of frozen tissue sections of lung were shown. Scale bars, 2000 μm; 400 μm. **c** qRT-PCR results of 25 cytokines expression in GC cell line MGC803 and CAFs were compared. **d**, **e** HGF mRNA and protein expression levels in normal gastric epithelial cell line GES-1, GC cell lines and three pairs of CAFs and NFs were quantified by qRT-PCR and ELISA (36 h after change the culture medium), respectively

CAFs and their normal counterparts were isolated from GC tissues and corresponding non-cancerous tissues, respectively. Immunofluorescent staining identified spindle-shape fibroblasts by vimentin and CAFs were verified by enhanced-expression of α-SMA and FAP (Fig. 1a). The markers of epithelial cells, endothelial cells and leukocytes were also used to insure the purity of fibroblasts (Supplement Fig. 1A). An animal model was built to assess the contribution of CAFs to the migration of GC cells. CAFs labeled with Dil were injected into athymic nude mice through caudal vein, followed by MGC803 cells labeled with DiO a week later. The mice were sacrificed in 1 week and lung tissues were examined. In most cases, MGC803 cells assembled at the same place where CAFs settled down (Fig. 1b). The result suggests existing cytokines derived from CAFs but not from MGC803 cells. To identify these factors, a number of cytokines mainly from stromal cells but rarely from epithelial cells were subjected to quantitative real-time PCR (qRT-PCR) in MGC803 cells and CAFs. As shown in Fig. 1c, HGF mRNA level was significantly higher in CAFs compared with MGC803 cells. To confirm the dominant driver of HGF, one immortalized normal gastric epithelial cell line GES-1, 12 human GC cell lines and three pairs of primary fibroblasts were subjected to qRT-PCR and enzyme-linked immunosorbent assay (ELISA). HGF was mainly expressed in fibroblasts, especially in CAFs (Fig. 1d, e). In addition, the mRNA expression of HGF and α-SMA were examined in 35 pairs of GC and corresponding adjacent non-cancerous gastric tissues. Both HGF and α-SMA mRNA expression levels were significantly higher in GC tissues (Supplement Fig. 1B), and HGF was positive correlated with α-SMA on mRNA level (Supplement Fig. 1C). In conclusion, these results suggested that HGF was predominantly expressed in CAFs and may serve some distinct functions in GC progression.

### HGF from CAFs promotes *MET*-unamplified GC proliferation and migration in vitro

Immunofluorescence stain of c-Met, the only known receptor of HGF, in GC tissues and adjacent non-cancerous gastric tissues showed that c-Met expression was higher in GC tissues than normal tissues, and higher in GC cells than fibroblasts (Supplement Fig. 2A). An online database of Gene Expression across Normal and Tumor tissues (GENT) containing more than 21,000 samples was used to confirm the higher expression of *MET* gene in tumor tissues, especially in GC tissues (Supplement Fig. 2B). Furthermore, analyzing a platform of 20,981 tumor samples from The Cancer Genome Atlas (TCGA) in cBioportal Web resource online (cBioportal for Cancer Genomic) revealed that the amplification of *MET* gene accounted for a considerable part of alterations, especially in GC (Supplement Fig. 2C). In addition, *MET* gene alteration was correlated with disease-free survival but not with overall survival (Supplement Fig. 2D).

**Fig. 2:**
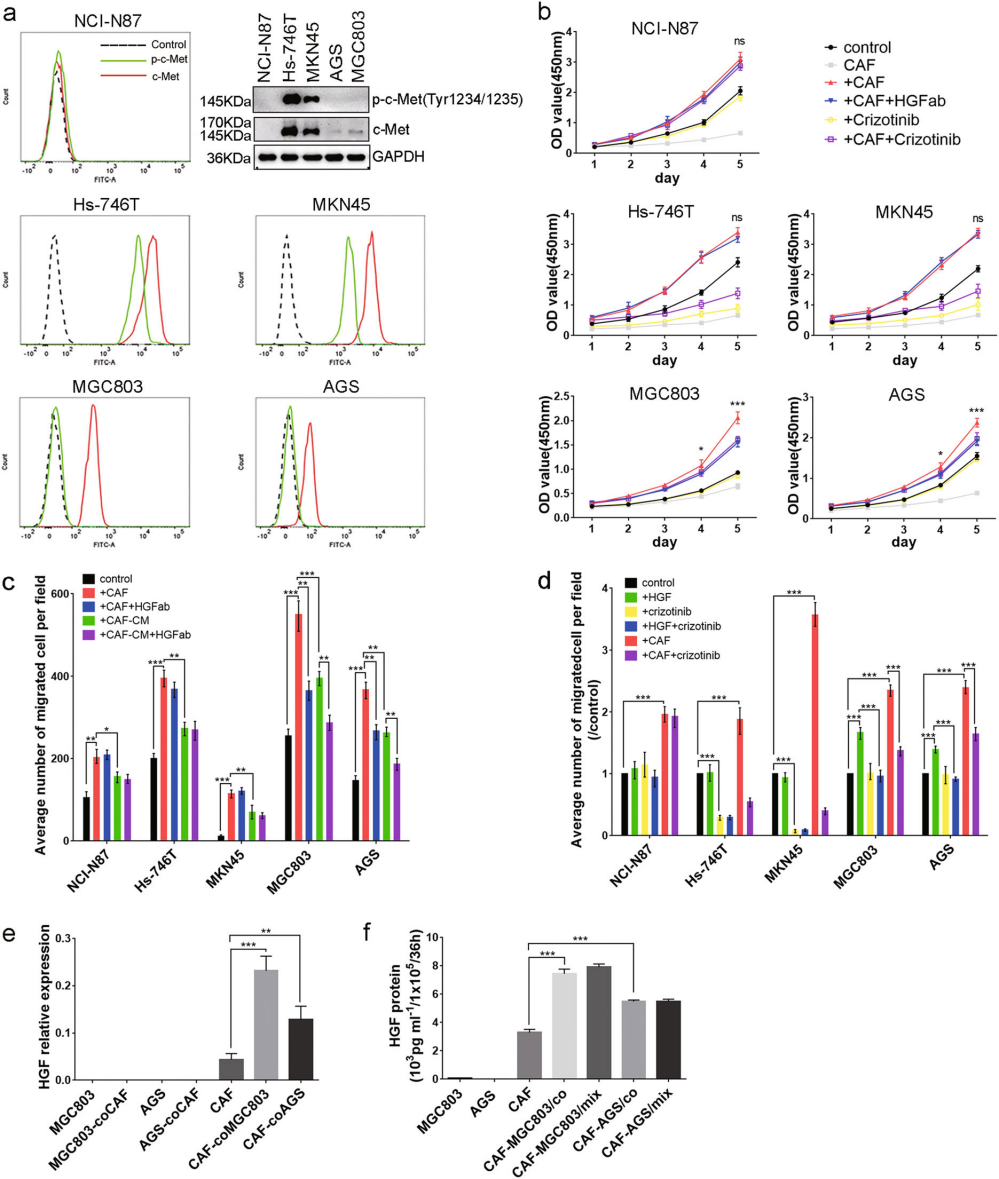
CAFs-derived HGF is associated with enhanced cell proliferation and migration of *MET*-unamplified GC. **a** Baseline expression of c-Met and p-c-Met in non-*MET* GC cell line NCI-N87, *MET*-amplified GC cell lines Hs-746T and MKN45, and *MET*-unamplified GC cell lines MGC803 and AGS were measured by western blot and FC. Lines and areas were used to indicate protein expression: black dotted lines, stained with isotype-control IgG; green solid lines, p-c-Met; red solid lines, c-Met. **b** CAFs-derived HGF promoted the proliferation of *MET*-unamplified GC cells. In the groups with crizotinib, GC cells were pretreated with crizotinib for 6 h before they were mixed with CAFs. **c**, **d** CAFs-derived HGF promoted the migration of MET-unamplified GC cells through c-Met. In the groups with crizotinib, GC cells were pretreated with crizotinib for 6 h before the transwell assays. **e**, **f** HGF mRNA expression and protein levels in MGC803, AGS, and CAFs were measured by qRT-PCR and ELISA. HGF (50 ng/ml); HGFab (300 ng/ml); Crizotinib (0.1 Μm). (ns, no significant difference; *P < 0.05; **P < 0.01; ***P < 0.001)

GC cell lines were classified into non-*MET*, *MET*-amplified, and* MET*-unamplified according to copy number of oncogene *MET* as described in previous study^27^. NCI-N87 was selected as non-MET, Hs-746T and MKN45 as *MET*-amplified, and MGC803 and AGS as *MET*-unamplified according to the expression of c-Met and phospho-c-Met^(Tyr1234/1235)^ proteins (Fig. 2a). To evaluate the effect of HGF derived from CAFs on proliferation of GC, an appropriate concentration of c-Met inhibitor crizotinib was found out (Supplement Fig. 3). As shown in Fig. 2b, NCI-N87 without c-Met expression showed no difference with or without HGF or crizotinib existed. The *MET*-amplified GC, Hs-746T and MKN45, showed no response to HGF but were highly sensitive to crizotinib. However, *MET*-unamplified GC, MGC803 and AGS, showed lower baseline proliferation with HGF neutralization or c-Met inhibition. CAFs promoted migration of all cell lines in a co-culture system, but only *MET*-unamplified GC were sensitive to CAFs-derived HGF (Fig. 2c). Recombinant human HGF protein promoted migration of *MET*-unamplified GC cells but not non-*MET* or *MET*-amplified GC cells (Fig. 2d). The above results suggested that only *MET*-unamplified GC cells were involved in the influence of CAFs-derived HGF on cell proliferation and migration. It was interesting that a co-culture system were more powerful than CAFs condition medium in facilitating migration (Fig. 2c). Thus, the expression of HGF in MGC803 cells, AGS cells and CAFs were examined. CAFs enhanced HGF mRNA and protein expression when co-cultured with MGC803 cells and AGS cells (Fig. 2e, f). However, it made no differences in HGF secretion between co-culture system and direct contact system (Fig. 2f). These data emphasize the importance of CAFs in tumor microenvironment and indicate that CAFs-derived HGF specially promote *MET*-unamplified GC progression.

### The tumor-promoting effects induced by CAFs-derived HGF on *MET*-unamplified GC cells are mediated through the activation of ERK1/2 and STAT3 signaling pathways

**Fig. 3:**
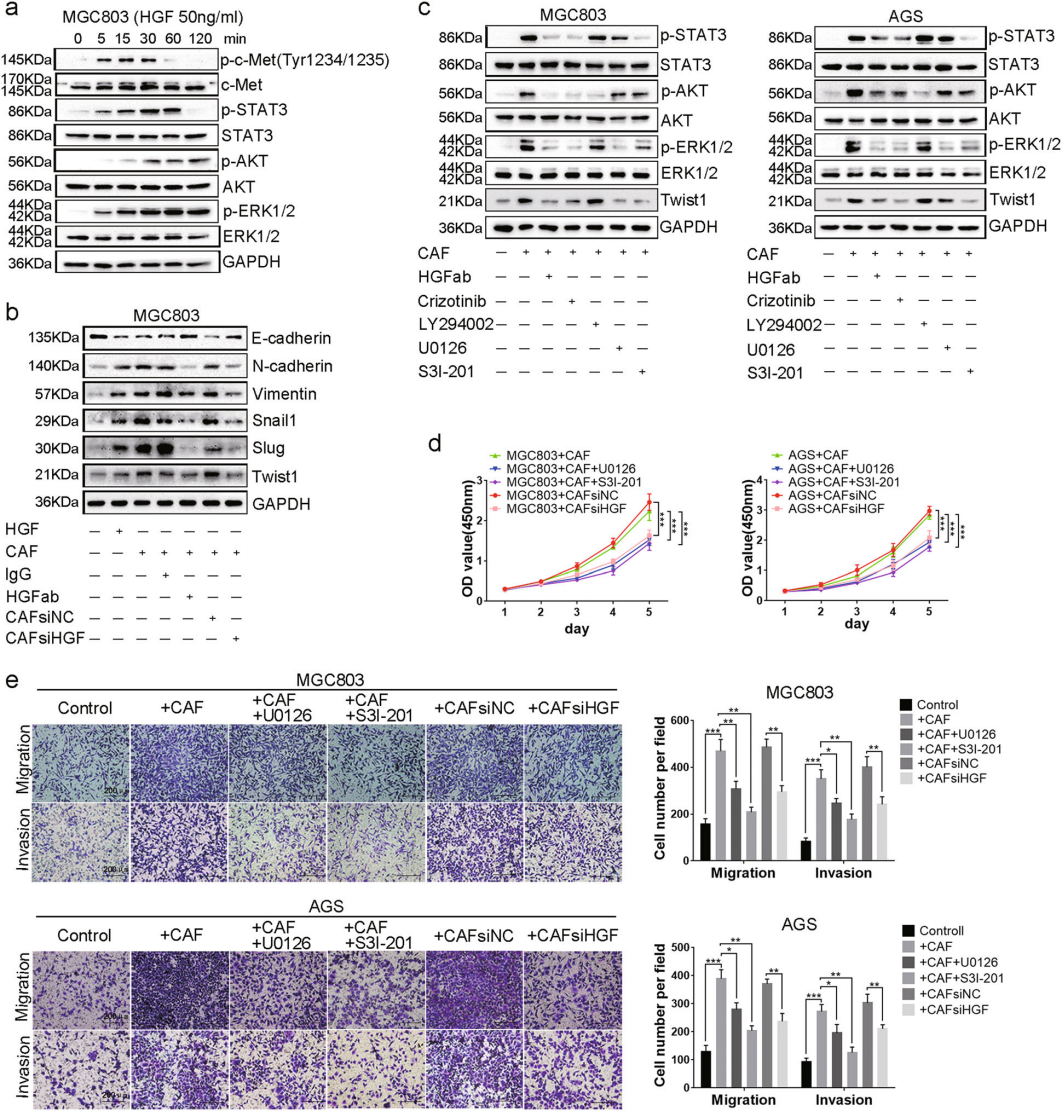
CAFs-derived HGF induces EMT and promotes proliferation, migration, and invasion of *MET*-unamplified GC cells via ERK1/2 and STAT3 signaling. **a** Downstream oncogenic signals triggered by HGF in MGC803 cells. **b** Expression of EMT markers in MGC803 cells were detected by western blotting. MGC803 cells were lysed after treatment with recombinant human HGF protein for 2 days or co-cultured with CAFs for 2 days. **c** Twist1 expression in MGC803 and AGS cells were detected by western blotting. GC cells were pretreated with inhibitors for 6 h, and the same concentration of these inhibitors were added into co-culture system. **d** Schematic charts of cell growth were measured by CCK-8. GC cells were pretreated with inhibitors for 6 h before they were mixed with CAFs. **e** Cell migration and invasion of MGC803 and AGS cells with different treatments as indicated were determined using transwell assays. Scale bars, 200 μm. HGF (50 ng/ml); HGFab (300 ng/ml); Crizotinib (0.1 Μm); LY294002 (50 μM); U0126 (20 μM); S3I-201 (100 μM). (*P < 0.05; **P < 0.01; ***P < 0.001)

HGF bound to c-Met and then triggers a number of downstream oncogenic signaling cascades such as PI3K/AKT, ERK1/2, and p-STAT3 in *MET*-unamplified GC, MGC803 and AGS cells (Fig. 3a and Supplement Fig. 4A). Gene set enrichment analysis (GSEA) using RNA-seq of 415 GC samples from TCGA and microarray profiles of 300 GC samples from GSE62254 showed that HGF was highly correlated with epithelial-mesenchymal transition (EMT) (data not shown). Then, we examined the change of typical markers of EMT. Both treated with recombinant human HGF protein and co-culture with CAFs markedly decreased epithelial marker (E-cadherin) expression and increased mesenchymal markers (N-cadherin, Vimentin, Snail, Slug, and Twist1) expression in MGC803 and AGS cells (Fig. 3b and Supplement Fig. 4C). When HGF was inhibited, whether by using HGF neutralizing antibody or small-interfering RNA (Supplement Fig. 4B), EMT induced by CAFs was impaired (Fig. 3b and Supplement Fig. 4C). As twist1 protein was known to promote tumor metastasis by inducing invadopodia formation^28^, we analyzed databases of TCGA and GSE62254 and found a positive correlation between HGF and twist1 (Supplement Fig. 4D, E). To investigate how CAFs-derived HGF influenced twist1 expression, MGC803 and AGS cells were co-cultured with CAFs and downstream signals of HGF/c-Met were inhibited using corresponding inhibitors. Twist1 expression was significantly inhibited when treated with MEK1/2 inhibitor, U0126, and STAT3 inhibitor, S3I-201, but not with PI3K/AKT inhibitor, LY294002 (Fig. 3c). To confirm whether the ERK1/2 and STAT3 signaling pathways participated in HGF-enhanced proliferation, migration and invasion, corresponding experiments were conducted. Cell proliferation of MGC803 cells and AGS cells were significantly inhibited when HGF expression in CAFs was decreased (Fig. 3d). Meanwhile, cell proliferation also dramatically impaired after treatment with U0126 and S3I-201 compared with untreated groups (Fig. 3d). In addition, the inhibition of HGF expression as well as the inhibitors of ERK1/2 and STAT3 signaling pathways significantly reduced migrated and invaded MGC803 and AGS cells (Fig. 3e). Altogether, these in vitro data suggest that CAFs-derived HGF increases twist1 expression in *MET*-unamplified GC cells and shows biological function via activating ERK1/2 and STAT3 signaling pathways.

### Positive cytokine-receptor interactions between *MET*-unamplified GC and CAFs increases twist1 expression and shows radical promotion of GC progression in vitro

**Fig. 4:**
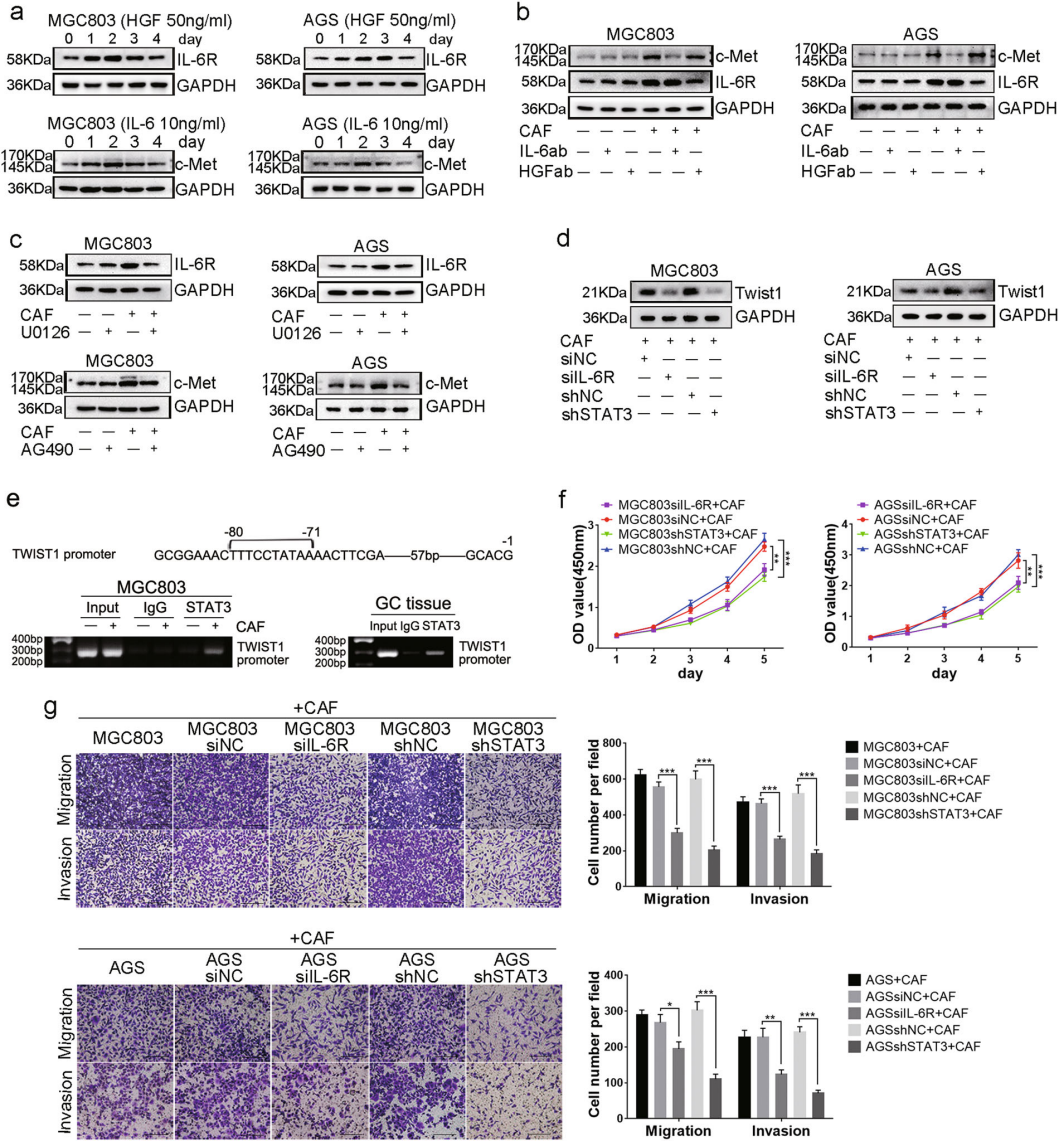
Cytokine-receptor interactions between *MET*-unamplified GC cells and CAFs promote GC cells progression via STAT3 signaling. **a** HGF induced IL-6R expression, and IL-6 induced c-Met expression in MGC803 and AGS cells in time gradient. **b** IL-6R and c-Met expression in co-culture system were detected by western blotting. GC cells and CAFs were co-cultured for 2 days. **c** HGF increased IL-6R expression via ERK1/2 signaling, and IL-6 increased c-Met expression via JAK2 signaling. GC cells were pretreated with inhibitors for 6 h, and the same concentration of these inhibitors were added into co-culture system. **d** Twist1 expression in GC cells transfected with shRNA or siRNA were detected by western blotting. **e** ChIP assays performed in MGC803 cells and in GC tissues. **f** Cell proliferation of MGC803 and AGS cells were measured by CCK-8 assays. **g** Migration and invasion of MGC803 and AGS cells were measured by transwell assays. Scale bars, 200 μm. HGF (50 ng/ml); HGFab (300 ng/ml); IL-6 (10 ng/ml); IL-6ab (150 ng/ml); U0126 (20 μM); AG490 (10 μM). (*P < 0.05; **P < 0.01; ***P < 0.001)

Given above results, it is confused that only S3I-201 decreased twist1 expression induced by recombinant human HGF (Supplement Fig. 5A). When looking back, we found that ERK1/2 signal inhibition induced STAT3 signal inhibition; meanwhile, STAT3 signal inhibition also induced ERK1/2 signal inhibition (Fig. 3c). It is conceivable that there is a crosstalk between GC cells and CAFs, which indicates the complexity of tumor microenvironment. As STAT3 signal was always activated by IL-6/JAK2 signaling pathway, the expression of IL-6 mRNA and protein in MGC803 cells, AGS cells and CAFs were examined. IL-6 was mainly expressed in CAFs and upregulated when in co-culture system (Supplement Fig. 5B). Immunofluorescence staining of IL-6 and IL-6R in GC tissues and adjacent non-cancerous gastric tissues showed that IL-6 was mainly expressed in fibroblasts, while IL-6R in both cancer cells and fibroblasts (Supplement Fig. 6A, B). Considering this, we suppose that CAFs-derived HGF-enhanced IL-6/JAK2/STAT3 signaling pathway in GC cells when co-cultured with CAFs (in the presence of IL-6), but not when cultured GC cells alone (in the absence of IL-6). To verify the idea, GSEA and correlation analyses were performed and the results showed a positive correlation between HGF and IL-6R (Supplement Fig. 5C, D). The regulation of IL-6R by HGF was investigated next. As shown in Fig. 4a, recombinant human HGF protein increased the expression of IL-6R in both MGC803 and AGS cells. We also found that IL-6 could increase c-Met expression, which also had been reported in myeloma cells^29^. A co-culture system was then built to confirm above results. As shown in Fig. 4b, co-cultured with CAFs increased the expression of c-Met and IL-6R in GC cells, which were reversed by IL-6 neutralization and HGF neutralization, respectively. To investigate the mechanism how HGF regulated IL-6R expression, a MEK1/2 inhibitor, U0126, was used to inhibit ERK1/2 signaling. U0126 decreased IL-6R expression in MGC803 and AGS cells in co-culture system (Fig. 4c). Previous studies has shown that IL-6/JAK2/STAT3 axis played an active role in oncogenesis of tumors^22,26,30^. To avoid the interference of p-STAT3 induced by HGF/c-Met signaling, a JAK2 inhibitor, AG490, was applied as IL-6/JAK2/STAT3 signaling inhibitor. AG490 decreased c-Met expression in MGC803 and AGS cells when they were co-cultured with CAFs (Fig. 4c). Above results suggested that CAFs-derived HGF increased IL-6R expression in* MET*-unamplified GC cells through HGF/c-Met/ERK1/2 signaling pathway, and that CAFs-derived IL-6 increased c-Met expression in* MET*-unamplified GC cells through IL-6/IL-6R/JAK2/STAT3 signaling pathway.

To elucidate the influence of IL-6R and STAT3 on twist1 expression, IL-6R and STAT3 expressions were silenced by transfecting IL-6R small-interfering RNA (siRNA) and STAT3 short hairpin RNA (shRNA) into MGC803 and AGS cells (Supplement Fig. 5E, F). In co-culture system, twist1 expression decreased in GC cells when transfected with IL-6R siRNA or STAT3 shRNA (Fig. 4d). Immunofluorescence staining of p-STAT3 and twist1 in MGC803 cells indicated their co-expression on the condition of CAFs-derived HGF (Supplement Fig. 5G). Next, *TWIST1* promoter region for potential STAT3-binding sites was analyzed using the JASPAR database and ALGGEN-PROMO, and the result was consistent with previous study^31^. Then chromatin immunoprecipitation assays were performed in Both MGC803 cells and GC tissues. As indicated in Fig. 4e, CAFs activated the binding ability of p-STAT3 to STAT3-binding site (–71 to –80 relative to the transcription start site) in the *TWIST1* promoter. Function studies were performed to further confirm the biological roles of CAFs-derived HGF via IL-6R and STAT3. Cell proliferation, migration, and invasion of *MET*-unamplified GC cells was significantly inhibited when IL-6R and STAT3 expression decreased (Fig. 4f, g). Taking together, we hypothesize that CAFs-derived HGF induce twist1 expression not only via activating HGF/c-Met/STAT3 but also by enhancing IL-6/IL-6R/JAK2/STAT3 signaling pathway through increasing IL-6R expression, and CAFs-derived IL-6 intensify the promoting effects of HGF by increasing c-Met expression, thus building positive cytokine-receptor interactions between *MET*-unamplified GC cells and CAFs. And therefore, the cooperation accelerates *MET*-unamplified GC progression.

### HGF enhances IL-6-induced transdifferentiation of quiescent fibroblasts to CAFs

**Fig. 5:**
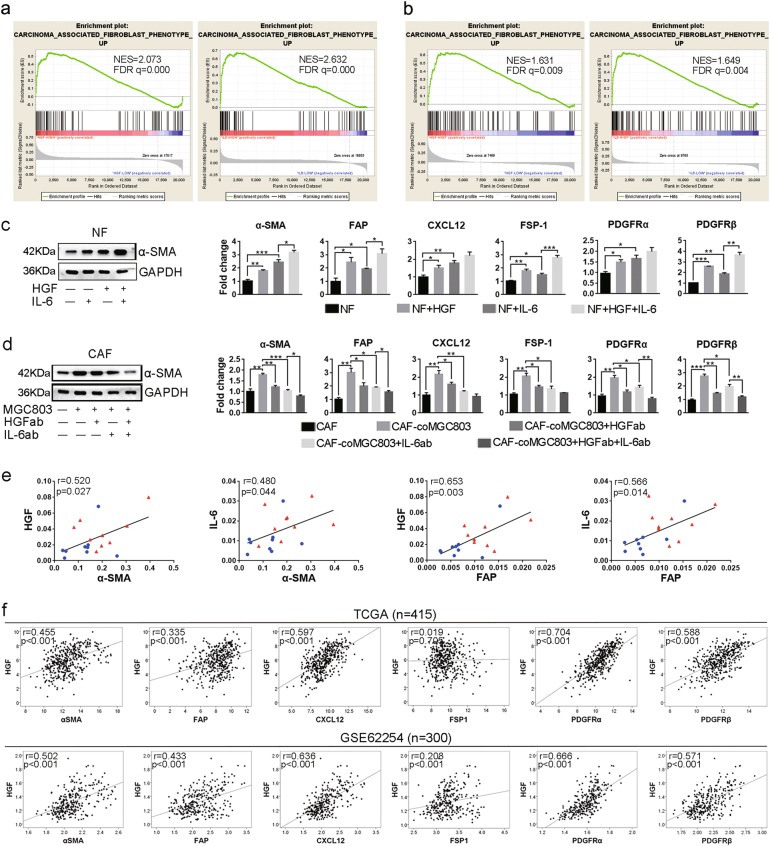
HGF facilitates IL-6-enhanced characteristic of CAFs. **a** Gene set enrichment analyses (GSEA) of GC samples from TCGA showed that high HGF (left) and IL-6 (right) expression were positively associated with upregulation of carcinoma-associated fibroblasts phenotype. Each bar corresponds to one gene. **b** GSEA results of HGF (left) and IL-6 (right) in GC samples from GSE62254. **c** HGF and IL-6 increased CAFs markers expression in NFs. NFs were lysed after treatment with HGF and IL-6 for 2 days. **d** HGF and IL-6 neutralization decreased CAFs markers expression in CAFs. **e** HGF and IL-6 were positively correlated with α-SMA and FAP in nine pairs of CAFs (red solid triangle) and NFs (blue solid circle), respectively. **f** Positive correlation of HGF and CAFs markers were analyzed with samples from TCGA and GSE62254. HGF (50 ng/ml); HGFab (300 ng/ml); IL-6 (10 ng/ml); IL-6ab (150 ng/ml). (**P < 0.01; ***P < 0.001)

Accumulating evidences suggested that HGF and IL-6 contributed to CAFs activation^8,12,32^. GSEA of two independent GC patients’ databases from TCGA and GSE62254 showed that high HGF and IL-6 expression were associated with upregulation of genes related to CAFs phenotype (Fig. 5a, b). As shown in Fig. 5c, HGF and IL-6 increased protein expression of α-SMA and mRNA expression of the markers of CAFs (α-SMA, FAP, CXCL12, FSP-1, PDGFRα, and PDGFRβ) in NFs. Moreover, HGF-enhanced IL-6-induced expression of α-SMA, FAP, FSP-1, and PDGFRβ in NFs (Fig. 5c). In co-culture system, *MET*-unamplified GC cells increased the expression of CAFs markers, which were reversed by both HGF neutralization and IL-6 neutralization (Fig. 5d and Supplement Fig. 7A).

To further verify the correlation between HGF, IL-6 and characteristic of CAFs, the mRNA expression of HGF, IL-6, and CAFs markers, α-SMA and FAP, in nine paired CAFs and NFs were examined. Both HGF and IL-6 were found to be positively correlated with α-SMA and FAP, respectively (Fig. 5e). In addition, correlation analysis showed that both HGF and IL-6 were positively correlated with CAFs markers (Fig. 5f and Supplement Fig. 7B). Activated fibroblasts are functionally distinguishable from their homologously quiescent fibroblasts^33,34^. Both HGF and IL-6 facilitated cell migration of NFs, and the number of migrational NFs induced by IL-6 has significantly increased by HGF (Supplement Fig. 7C). Meanwhile, co-culture with *MET*-unamplified GC cells promoted cell migration of CAFs, which was reversed by both HGF neutralization and IL-6 neutralization (Supplement Fig. 7D). These observations suggested that both HGF and IL-6 participate in transdifferentiation of NFs to CAFs, and HGF could enhance the promoting effect of IL-6. So the cooperation of HGF and IL-6 influence not only *MET*-unamplified GC cells but also fibroblasts.

### CAF-derived HGF promotes* MET*-unamplified GC tumorigenesis and metastasis through STAT3 signaling in vivo

**Fig. 6:**
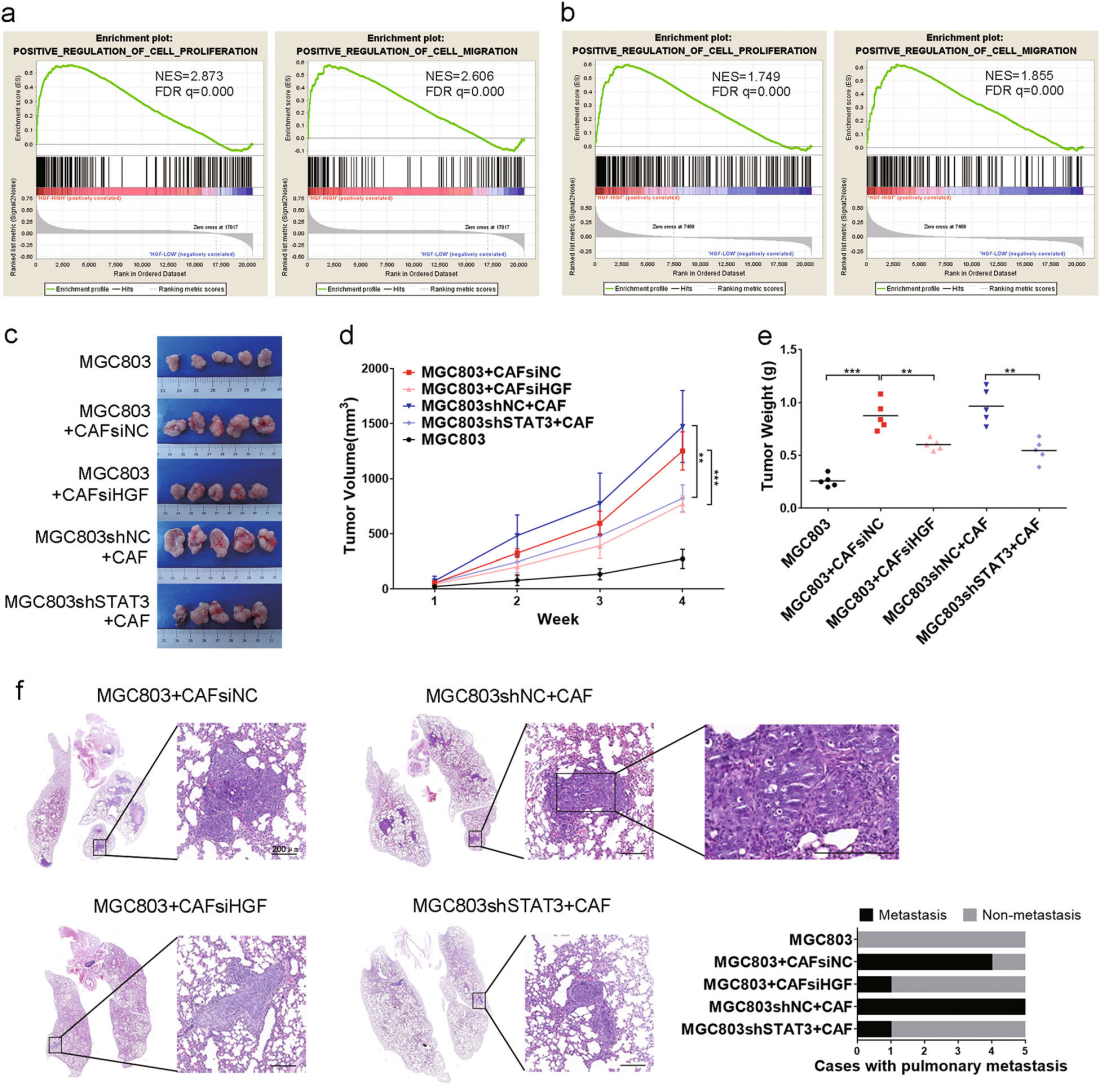
Decreased HGF expression in CAFs and STAT3 expression in *MET*-unamplified GC cells impair tumorigenesis and metastasis. **a** GSEA results of GC samples from TCGA showed that high HGF expression was positively correlated with increased cell proliferation and migration. **b** GSEA results of HGF in GC samples from GSE62254. **c** Representative image of tumor mass for each group. **d** Tumor growth curves of tumor volume. **e** Tumor weight of each group. **f** Representative images of pulmonary metastasis and graph of cases with pulmonary metastasis in each group. Scale bars, 200 μm. (**P < 0.01; ***P < 0.001)

**Fig. 7:**
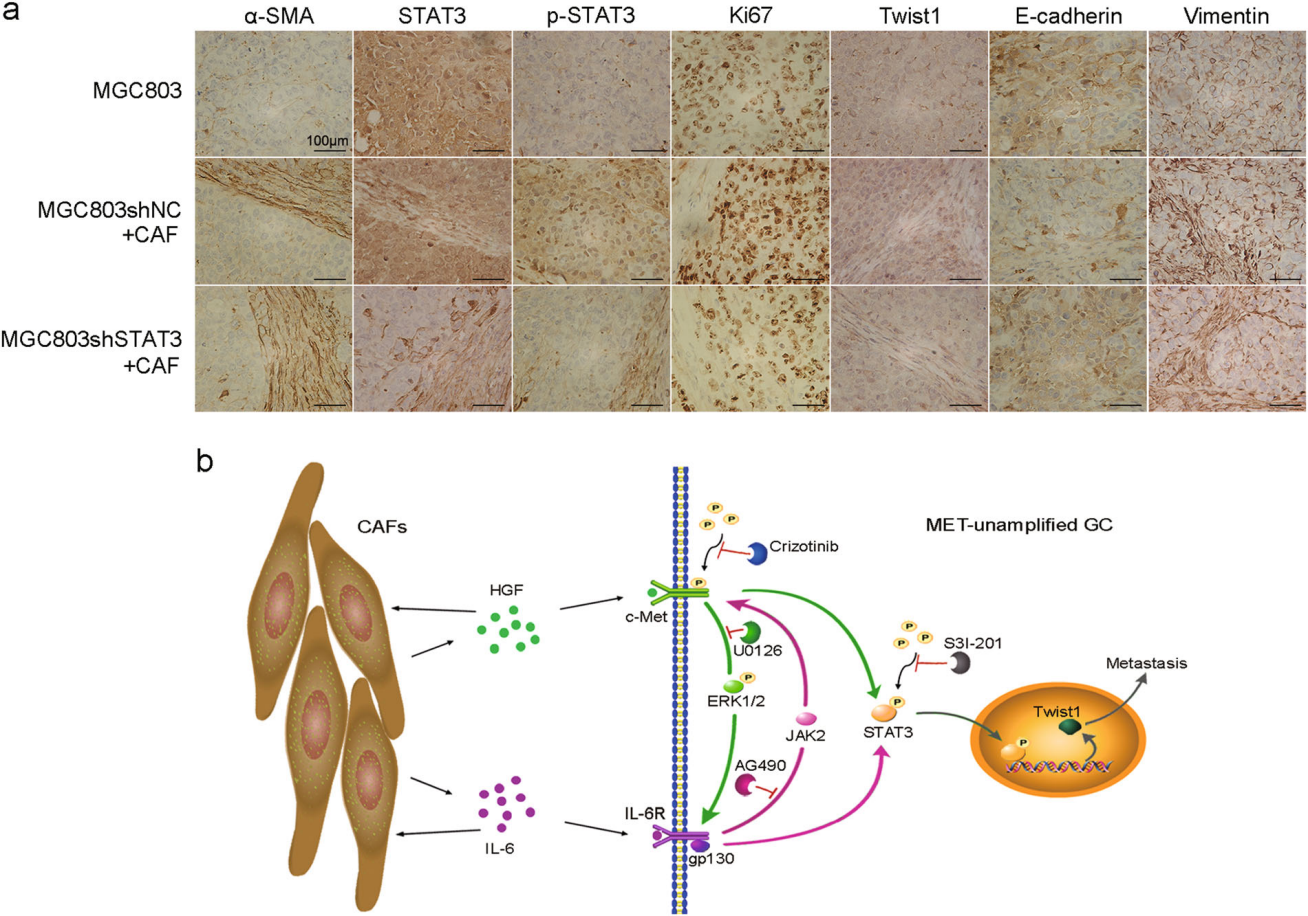
Immunohistochemistry staining of tumors from nude mice and schematic diagram showing the effects of CAFs-derived HGF and IL-6 on *MET*-unamplified GC cells. **a** Immunohistochemistry staining of tumors showed that CAFs increased the expression of Ki67, twist1, and vimentin, as well as decreased expression of E-cadherin in MGC803 cells, which were reserved by inhibiting STAT3 expression. Scale bars, 100 μm. **b** Schematic diagram showed that the cooperation of HGF and IL-6 enhanced the characteristic of CAFs and promoted twist1 expression via STAT3 signaling in* MET*-unamplified GC cells

The promoting effects of HGF on cell proliferation and migration were confirmed by GSEA with databases of GC samples from TCGA and GSE62254, respectively (Fig. 6a, b). The functions of CAFs-derived HGF on *MET*-unamplified GC tumorigenesis and metastasis were evaluated in vivo. Co-injection of MGC803 cells and CAFs showed progressive growth than MGC803 cells alone (Fig. 6c, d). However, inhibition of HGF expression in CAFs and STAT3 in MGC803 cells significantly decreased tumor growth (Fig. 6c, d). Additionally, co-injection of MGC803 cells and CAFs significantly increased the average weight of tumors as compared to MGC803 cells alone (Fig. 6e), which was reversed by both inhibition of HGF expression in CAFs (0.602 ± 0.062 g vs. 0.876 ± 0.256 g, *P* = 0.003) and inhibition of STAT3 expression in MGC803 cells (0.546 ± 0.156 g vs. 0.966 ± 0.196 g, *P* = 0.002) (Fig. 6e). Immunohistochemistry staining results showed that CAFs significantly increased Ki67, vimentin and twist1 expression, and decreased E-cadherin expression, which were reversed by inhibition of STAT3 expression (Fig. 7a). In addition, the incidence of pulmonary metastasis for MGC803 alone, MGC803 + CAFsiNC, MGC803 + CAFsiHGF, MGC803shNC + CAF, MGC803shSTAT3 + CAF were 0%, 80%, 20%, 100%, 20% under the vision of microscope, respectively (Fig. 6f). Likewise, the number and size of metastatic clusters in the MGC803 + CAFsiHGF and MGC803shSTAT3 + CAF groups were less than those of MGC803 + CAFsiNC and MGC803shNC + CAF groups, respectively (Fig. 6f). In addition, abundant fibroblasts were found in the metastatic clusters under microscope (Fig. 6f), which is consistent with the result of in vivo chemotaxis assay (Fig. 1b). Given the in vitro results above, we conclude that CAFs-derived HGF promotes tumorigenesis and metastasis of *MET*-unamplified GC in vivo, in part, via STAT3 signaling (Fig. 7b).

## Discussion

Stromal cells showed prominent roles in facilitating tumor progression through reciprocal interactions with neoplastic cells in tumor microenvironment^34–36^. However, the underlying mechanisms of interactions between tumor cells and the activated fibroblasts remain largely unexplored. In the current study, the chemotaxis of tumor cells induced by CAFs was evaluated in vivo by co-localization of Dil-labeled CAFs and DiO-labeled GC, and then the main cytokines secreted by CAFs and GC cells were compared. HGF and IL-6 were identified as critical cytokines in fibroblasts, especially CAFs. They collaborated with each other and showed functions of promoting *MET*-unamplified GC progression and facilitated transdifferentiation of quiescent fibroblasts to CAFs.

As the only known tyrosine kinase receptor of HGF currently, c-Met has shown its unlimited proto-oncogene potential in both HGF-dependent and independent manner in various solid tumors^37–39^. High expression of c-Met correlates with poor survival of GC patients and breast cancer patients^40,41^, and a series of inhibitors, such as crizotinib, and its antibodies have been applied to clinical practices. Though *MET* amplification accounts for only small part of total GC patients^42,43^, it is the most common of *MET* gene alteration, which leads to a poor disease-free survival in GC (Supplement Fig. 2C, D).* MET* amplification induces highly phosphorylated state of c-Met, which could activate several intracellular signaling pathways without HGF^18^. We tested whether HGF could change functional phenotype of GC cells with different state of c-Met and p-c-Met expression, and found that HGF only focused on *MET*-unamplified GC cells, which suggests that CAFs-derived HGF participates in communication with selective group of GC cells in tumor environment.

Twist1 is a basic helix-loop-helix domain-containing transcription factor that inducing EMT and promoting tumor metastasis^28,44,45^. HGF and IL-6 have been reported to induce EMT of tumor cells^46,47^. In the present study, we showed that CAF-derived HGF and IL-6 were upregulated in co-culture system and acted as positive regulators of twist1 expression. Further, we found that HGF induced the expression of IL-6R, and thus activated the IL-6/IL-6R/JAK2/STAT3/twist1 signaling pathway. We also found increased c-Met expression in *MET*-unamplified GC cells in response to recombinant human IL-6. These results indicate the important roles of HGF and IL-6 in complicatedly reciprocal interactions between CAFs and* MET*-unamplified GC cells.

Fibroblasts acquire malignancy phenotype when transformed to cancer-associated fibroblasts^48,49^. IL-6 is a stimulative factor that can accelerate this process^12,32^. Given CAFs are primary source of HGF and IL-6, in current study, CAFs marker expressions in NFs were increased when stimulated with HGF and IL-6. Meanwhile, enhanced-expression of these markers in CAFs induced by *MET*-unamplified GC cells was reversed by HGF neutralization and IL-6 neutralization. This indicates the cooperation work of HGF and IL-6 in transdifferentiation of quiescent fibroblasts to CAFs and in maintaining characteristic of CAFs.

Gene set enrichment analyses of GC samples from TCGA and GSE62254 showed that high HGF expression was positively correlated with tumor growth and metastasis, which were confirmed by both functional experiments of cell proliferation, migration, and invasion in vitro and in animal models. In summary, when *MET*-unamplified GC cells were co-cultured with CAFs, the HGF expression was significantly increased. Increased HGF intensified malignant phenotype of both *MET*-unamplified gastric cancer cells and CAFs, then CAFs with intensified malignant phenotype facilitated the expression of HGF, thus building a positive crosstalk. CAFs-derived HGF and IL-6 upregulated each other’s receptor in *MET*-unamplified GC cells and collaboratively facilitated phosphorylation of STAT3, thus promoting tumorigenesis and metastasis of *MET*-unamplified GC. However, how *MET*-unamplified GC cells promoted the expression of HGF in CAFs was still unclear, which required further study.

Our study linked HGF mediated crosstalk to the control of *MET*-unamplified GC progression, and factors participate in the crosstalk may serve as prognostic indicators and therapeutic targets. Crosstalks between tumor cells and stromal cells are essential for tumor progression. A better understanding of the underlying mechanisms accelerates the discovery of therapeutic interventions. It has been shown that anti-HGF has clinical benefits in a subgroup of pulmonary adenocarcinoma^50^. Thus, HGF-targeted therapy could be a possible approach in the treatment of *MET*-unamplified GC. However, extensive research is required before its application.

## Materials and methods

### Cell lines and primary cell isolation

Human GC cell lines SGC7901, BGC823, AGS, SNU-1, MKN45, MKN28, KATOIII, SNU-16, NCI-N87, HGC27, Hs-746T, MGC803, and GES-1 (an immortalized normal gastric epithelial cell line) were obtained from Shanghai Institute of Digestive Surgery, Shanghai, People’s Republic of China. Cells were cultured and passaged in RPMI-1640 medium supplemented with 10% fetal calf serum according to the manufacturer’s instructions. Primary fibroblasts were isolated from GC tissues and paired non-tumor tissues of nine independent GC patients, who underwent radical gastrectomy at the Department of Surgery, Ruijin Hospital, School of Medicine, Shanghai Jiao Tong University with informed consents. None of the patients received chemotherapy before surgery. GC tissues and paired non-tumor tissues were minced into organoids of 1 mm^3^ after gently swilled and seeded onto 10 cm petri dishes within 30 min after resection. Fibroblasts creep out and produced a homogenous fibroblastic cell population after 7 days of culture as described in our previous study^12^. To maintain the characters of primary cells, the subsequent experiments were performed using fibroblasts with up to ten passages.

### Reagents

Antibodies used to detect EMT including E-cadherin, N-cadherin, Vimentin, Snail1, Slug were purchased from Cell Signaling Technology. FAP, CD31, and CD45 antibodies from Santa Cruz Biotech, α-SMA antibody from Abcam and Pan-cytokeratin from Cell Signaling Technology were used to identify CAFs and paired NFs. Human HGF antibody and Human IL-6 antibody were purchased from R&D Systems and used for HGF and IL-6 neutralization, respectively. Crizotinib and U0126 from Cell Signaling Technology and S3I-201 from Abcam were used as inhibitors of HGF/c-Met signaling, ERK1/2 signaling and STAT3 signaling pathways, respectively. AG490 from Medchem Express used as IL-6/JAK2/STAT3 inhibitor. Recombinant human HGF protein from Abcam and recombinant human IL-6 protein from ABclonal Biotech were used as stimulating factors in the experiments, respectively. See Supplementary Table S1 for the reagents used.

### Gene set enrichment analysis (GSEA) and correlation analysis

RNA-seq of 415 GC patients from Stomach Adenocarcinoma (TCGA, Provisional) and microarray profiles of 300 GC patients from GSE62254 were downloaded from cBioPortal platform (http://www.cbioportal.org/) and GEO database (https://www.ncbi.nlm.nih.gov/geo/), respectively. GSEA 3.0 software (Broad Institute, Cambridge, MA, USA) was used for GSEA and the number of permutations was set to 1000. The mean value of each gene expression was used for correlation analysis.

### Plasmids transfection and RNA interference

STAT3 shRNA vectors and dominant-negative control were kind gifts from Mr. Yuan Ruosen (Renji hospital, Shanghai Jiao Tong University, Shanghai, China). MGC803 and AGS cells were cultured in 24-well plates and transfected with 2 μg shRNA plasmids with Lipofectamine 2000 reagent (Invitrogen, Carlsbad, CA). Stable transfected cells were selected with puromycin (1 μg/ml) (InvivoGen, San Diego, CA, USA) and used in the following experiments. Knockdown of STAT3 was validated by western blot. HGFsiRNA, negative controls, IL-6R siRNA and scrambled siRNA, were synthesized by Shanghai GenePharma, Co., Ltd., (Shanghai, China) and were chemically modified by 2’-O-Me. Control siRNA and targeted gene siRNA were transfected into corresponding cells with Lipofectamine 2000 reagent. See Supplementary Table S2 for siRNA and shRNA targeted sequences.

### Flow cytometry

Pan-cytokeratin, CD31 and CD45 in CAF and NF, c-Met and p-c-Met in GC were measured as by Flow cytometry (FC). Briefly, equal cells were collected and permeated with 0.5% Triton for 10 min, washed with PBS and then stained with primary antibody at 4 °C overnight. Cells were then incubated with Alexa Fluor 488 dye-conjugated secondary antibody at room temperature for 3 h in the dark after being washed with PBS for three times. The fluorescence intensity of Pan-cytokeratin, CD31 and CD45 in CAF and NF, c-Met and p-c-Met in GC was detected by Flow Cytometer and the data were analyzed by FlowJo V10 software.

### Immunofluorescence

Briefly, cells and the frozen sections of tissue samples were fixed in 1% neutralized formaldehyde buffer for 30 min at room temperature, followed by permeabilization with 0.5% Triton for 10 min. Cells were blocked with 3% bovine serum albumin, sections with normal non-immune goat serum, both for 30 min at room temperature. After being gently washed three times with PBS, cells and sections were incubated at 4 °C overnight with primary antibodies for α-SMA, FAP, Vimentin, HGF, MET, IL-6, IL-6R, p-STAT3, Twist1 antibody. Cells and sections were stained with appropriate Alexa dye-conjugated secondary immune reagents at room temperature in the dark after three PBS washes. Then cells and sections were subjected to an Olympus BX70 microscope (Olympus, Tokyo, Japan) after covered with slides with Anti-fade Reagent containing DAPI.

### Cell proliferation assay

Cell proliferation assay was performed using Cell Counting Kit-8 (Dojindo, Kumamoto, Japan). Briefly, 1 × 10^3^ GC or 1 × 10^3^ GC mixed with 3 × 10^2^ primary CAFs were suspended in 200 μl medium in 96-well plates with different treatments (rhHGF, HGFab, crizotinib, U0126 and S3I-201, concentrations shown in Supplementary Table S1) and incubated for 24, 48, 72, 96, and 120 h. After incubation, CCK-8 (20 μl) was added into each plate for 2 h at 37 °C. OD 450 was detected by spectrophotometry (BioTek, Vermont, USA). In the groups with inhibitors, GC cells were pretreated with inhibitors for 6 h before they mixing with CAFs, and then they were suspended in 200 μl medium in (96-well plates) containing the same concentration of these inhibitors.

### Cell migration and invasion assay

Cell migration and invasion assays were conducted using Matrigel (BD Bioscience, CA) in 8 μm transwell chambers (Corning Life Science, Acton, MA, USA). For GC cells migration assays, 5 × 10^4^ GC cells were suspended in 200 μl serum-free medium and cultured in the upper chamber for 17 h with or without 1.5 × 10^4^ fibroblasts in the lower chamber with 600 μl 10% serum-conditioned medium. In the groups with inhibitors, GC cells were pretreated with U0126 (20 μM) and S3I-201 (100 μM), respectively, for 6 h, and then suspended in 200 μl serum-free medium containing the same concentration of these inhibitors. Oppositely for the migration assays of NFs and CAFs. For invasion assays, 1 × 10^5^ GC cells were used as described above and cultured for 24 h after the inserts were coated with 50 μl Matrigel/well. Inserts were fixed in formalin and stained with 0.1% crystal violet for 30 min, then removed non-migrating or non-invading cells with cotton swabs. Nikon Digital Sight DS-U2 (Nikon, Tokyo, Japan) and Olympus BX50 microscopes (Olympus, Tokyo, Japan) were used to photograph migrated and invaded cells. Each experiment was performed three times on the same conditions.

### Enzyme-linked immunosorbent assay (ELISA)

The levels of cytokines HGF and IL-6 in supernatants of GC cells and fibroblasts were detected by ELISA kit (R&D Systems, Minneapolis, MN, USA) according to the manufacturer’s instructions. Briefly, GC cells (1 × 10^5^) and fibroblasts (1 × 10^5^) were culture alone or together with or without 0.4 μm-6-well plate transwell inserts (Millipore) in 2 ml of RPMI-1640 complete medium for 36 h. After centrifuging at 12,000 × *g* for 10 min to remove cell debris, cancer cell and fibroblasts conditioned medium as well as co-culture medium from the lower wells were collected for ELISA.

### Quantitative real-time PCR (qRT-PCR)

Total RNA extracted from cells and tissues using Trizol reagent (Invitrogen, Carlsbad, CA) was reversely transcribed to cDNA using a Reverse Transcription system (Promega, Madison, WI) according to the manufacturer’s instructions. The mRNA levels were quantified by qRT-PCR using the SYBR Green PCR Master Mix (Applied Biosystems, Waltham, MA, USA) ABI Prism 7900HT sequence detection system (Applied Biosystems, CA, USA). The relative mRNA levels were evaluated based on the Ct values and normalized to glyceraldehyde 3-phosphate dehydrogenase (GAPDH). The PCR primers for all genes are listed in Supplementary Table S2.

### Western blot analysis

In co-culture system, GC cells and CAFs were co-cultured for 2 days. GC cells were pretreated with inhibitors (crizotinib, LY294002, U0126, S3I-201 and AG490) for 6 h before co-cultured with CAFs in groups of inhibition, and the same concentration of these inhibitors were added into co-culture system for 2 days until cells were lysed in protein extraction reagent. Briefly, cells were lysed in mammalian protein extraction reagent (Pierce, Rockford, IL, USA) supplemented with protease and phosphatase inhibitor cocktail (Sigma-Aldrich, St. Louis, MO, USA). The same amount of protein samples were fractionated with 10% sodium dodecyl sulfate–polyacrylamide gel electrophoresis gel and then transferred onto 0.22 μm polyvinylidene fluoride (PVDF) membranes (Millipore, MA, USA). After blocking with 1 × TBST buffer supplemented with 5% bovine serum albumin at 37 °C for 2 h, the membranes were incubated at 4 °C overnight with the corresponding primary antibodies. The membranes were then incubated with HRP-conjugated secondary antibody (1:5000, LI-COR, Nebraska, USA) for 2 h at room temperature. Thermo Pierce chemiluminescent (ECL) Western Blotting Substrate (Thermo, Waltham, MA, USA) and infrared imaging system (LI-COR Biosciences, Lincoln, USA) were used to visualize the membranes. The antibodies used were shown in Supplementary Table S1.

### Chromatin immunoprecipitation (ChIP)

The ChIP assays were performed with Enzymatic Chromatin IP Kit (#9005, CST) according to the manufacturer’s instructions. Briefly, cells without treatment and co-cultured with cancer-associated fibroblast and GC tissues were cross-linked with 1% formaldehyde, and stopped by glycine. Cells were collected via centrifugation for 5 min at 4 °C, 1500 rpm. DNA was sheared by micrococcal nuclease to 150–900 bp. Nuclear membrane was broken by sonication. STAT3 antibody and normal IgG were used in Chromatin immunoprecipitation. After reversing the protein/DNA cross-links, PCR was performed to detect the sequences of *TWIST1* promoter. The product spanned extending from –45 to –329 regions of Twist1 promoter included putative binding sites. The primers used were shown in Supplementary Table S2.

### Immunohistochemistry staining (IHC)

Tissue samples were fixed with formalin and embedded with paraffin before slicing to 4 µm-thick slices and then Immunohistochemistry staining performed following EnVision two-step procedure of Dako REAL™ Envision™ Detection System (Dako, Agilent Technologies, Ca, USA). After antigen retrieval with 0.01 M citrate buffer (pH 6.0), samples were stained with the primary antibodies. Then samples were incubation in secondary antibody for 30 min at 37 °C and visualized with DAB solution, followed by counterstain with hematoxylin. The antibodies used were shown in Supplementary Table S1.

### In vivo tumorigenesis and metastasis

Four-week-old male healthy athymic nude mice received from the Institute of Zoology, Chinese Academy of Sciences were housed in a specific pathogen-free environment in the Animal Experimental Center, Ruijin Hospital, Shanghai Jiao Tong University School of Medicine. The experiments were reviewed and approved by the Institutional Review of Committee for Animal Use of the Shanghai Jiao Tong University. Mice were randomly divided into different groups (five mice per group) and then injected subcutaneously with MGC803 (2 × 10^6^) alone or accompanied by CAFs (5 × 10^5^) suspended in 100 µl PBS into flanks of the mice. Tumor size was measured weekly using digital Vernier caliper and tumor volume was calculated using the following formula: tumor volume = (Width^2^ × Length)/2. Mice were killed at 4 weeks and tumors were weighted and processed for immunohistochemical analysis. For the in vivo chemotaxis assay, five-week-old male healthy athymic nude mice were injected with Dil-labeled CAFs through caudal vein. One week later, the same mice were injected with DiO-labeled MGC803. After another week, mice were killed and the lungs were freezed and sliced to 200 µm-thick frozen tissues slices by Cryostat (Leica, Germany) and then subjected to Olympus BX70 microscope (Olympus, Tokyo, Japan) immediately. For pulmonary metastasis assay, nude mice were injected with CAFs (5 × 10^5^) or PBS (control) through caudal vein, followed by MGC803 (2 × 10^6^) in 1 week. Mice were killed at 8 weeks and lungs were collected and sliced to find metastasis focus.

### Statistical analysis

The statistical analysis was performed using GraphPad Prism6 and the experimental results were presented as mean ± standard deviation (SD). Differences between groups were compared by Student’s *t*-test and two-tailed *P*-value ≤ 0.05 was considered as significant.

## Electronic supplementary material

Supplementary Figure 1

Supplementary Figure 2

Supplementary Figure 3

Supplementary Figure 4

Supplementary Figure 5

Supplementary Figure 6

Supplementary Figure 7

Supplementary Table 1

Supplementary Table 2

Supplementary Figure Legends
