# Supplementary figures and images for: HGF-mediated crosstalk between cancer-associated fibroblasts and MET-unamplified gastric cancer cells activates coordinated tumorigenesis and metastasis
Source: Cell Death Dis. 2018 Aug 29;9(9):867. doi: 10.1038/s41419-018-0922-1 (PMC6115420; doi:10.1038/s41419-018-0922-1)

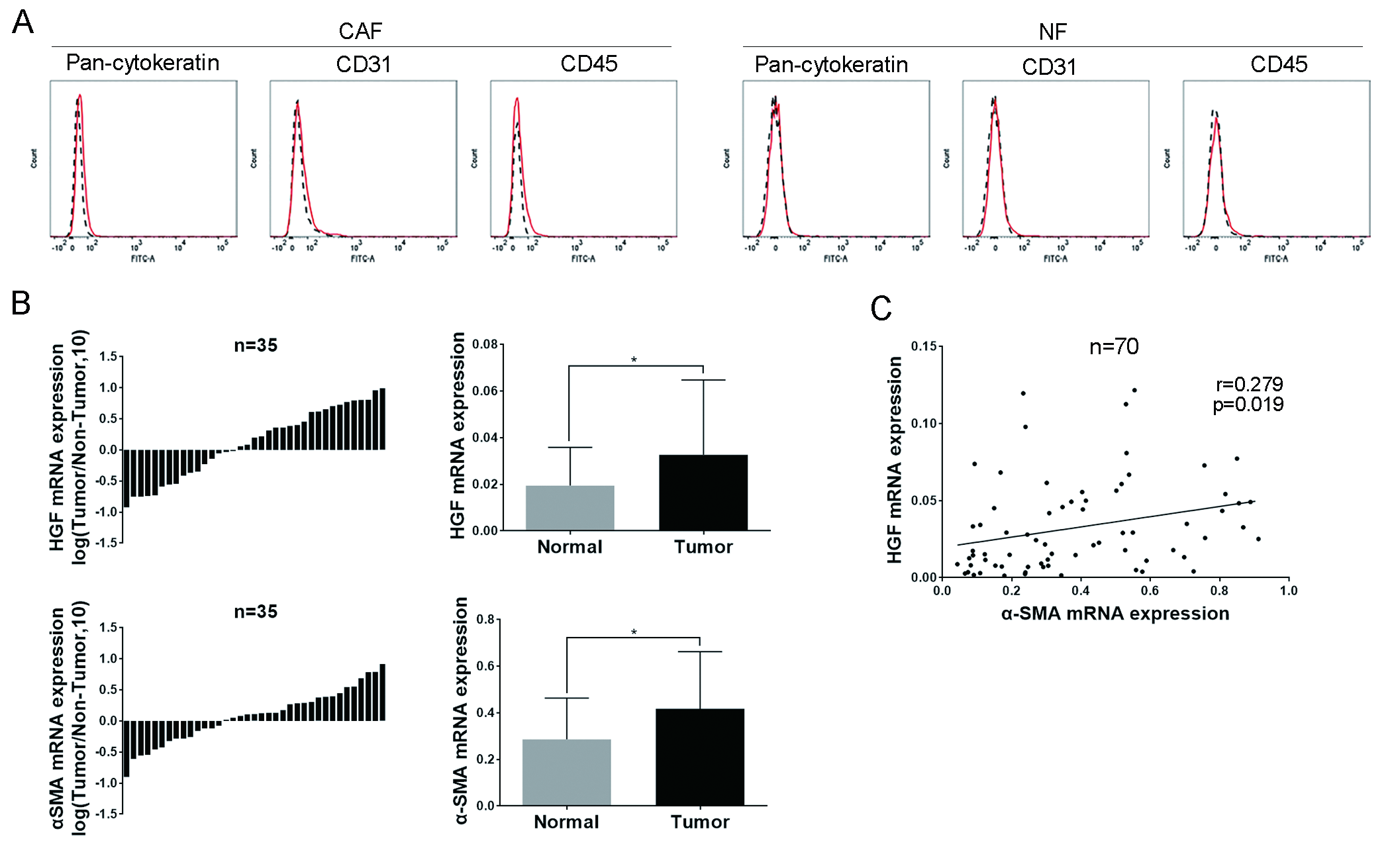

Supplement: Supplementary file 1 — Supplementary Figure 1 [file 41419_2018_922_MOESM1_ESM.tif]

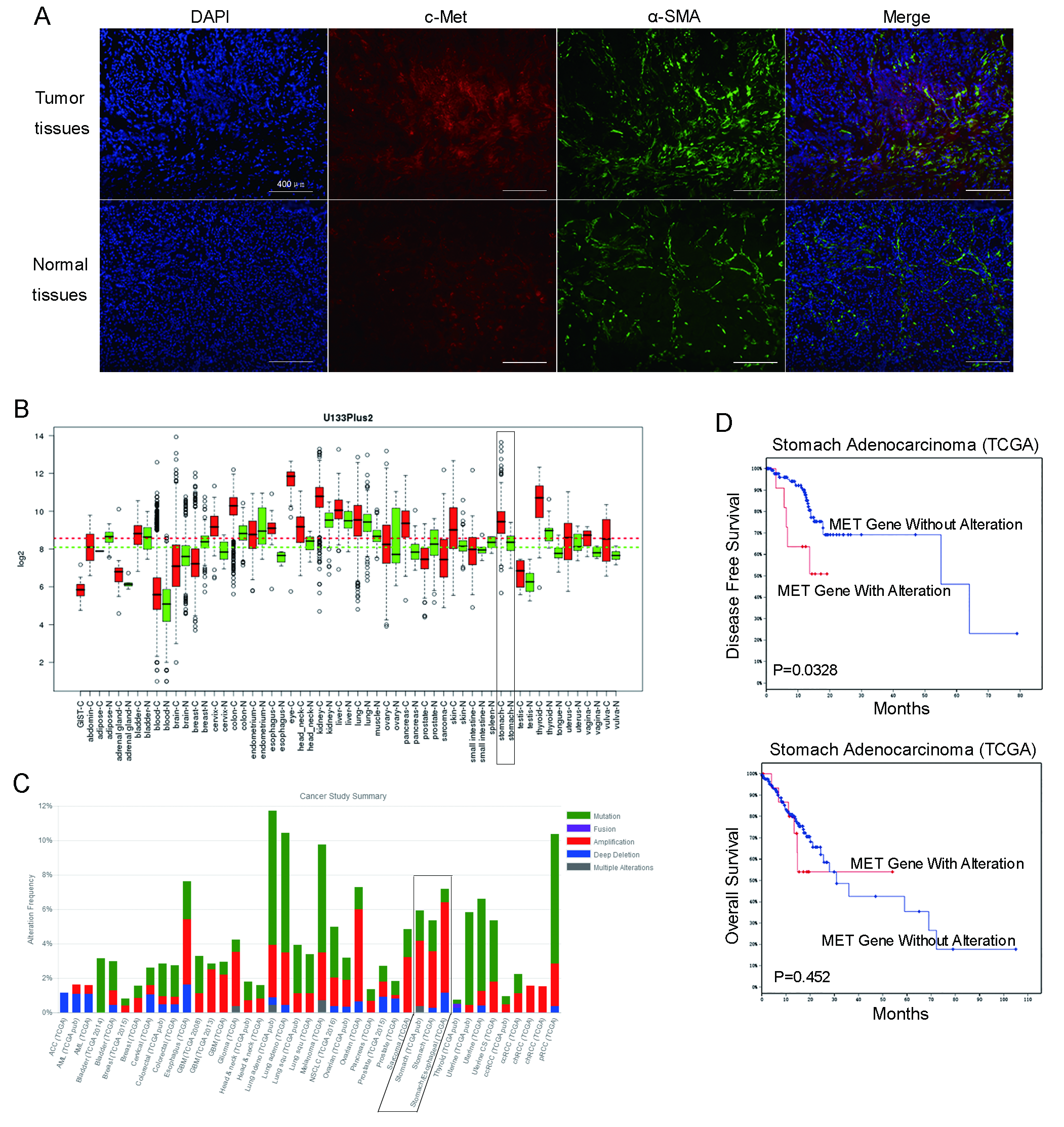

Supplement: Supplementary file 2 — Supplementary Figure 2 [file 41419_2018_922_MOESM2_ESM.tif]

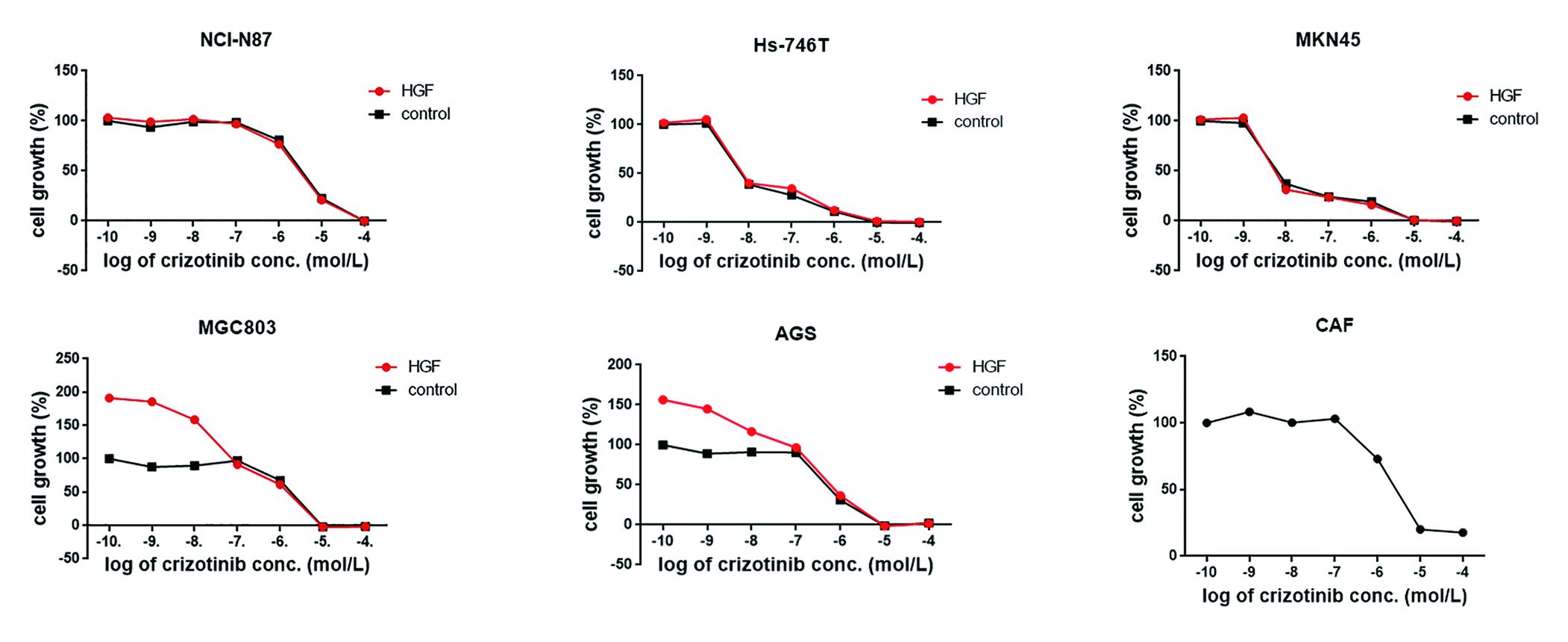

Supplement: Supplementary file 3 — Supplementary Figure 3 [file 41419_2018_922_MOESM3_ESM.tif]

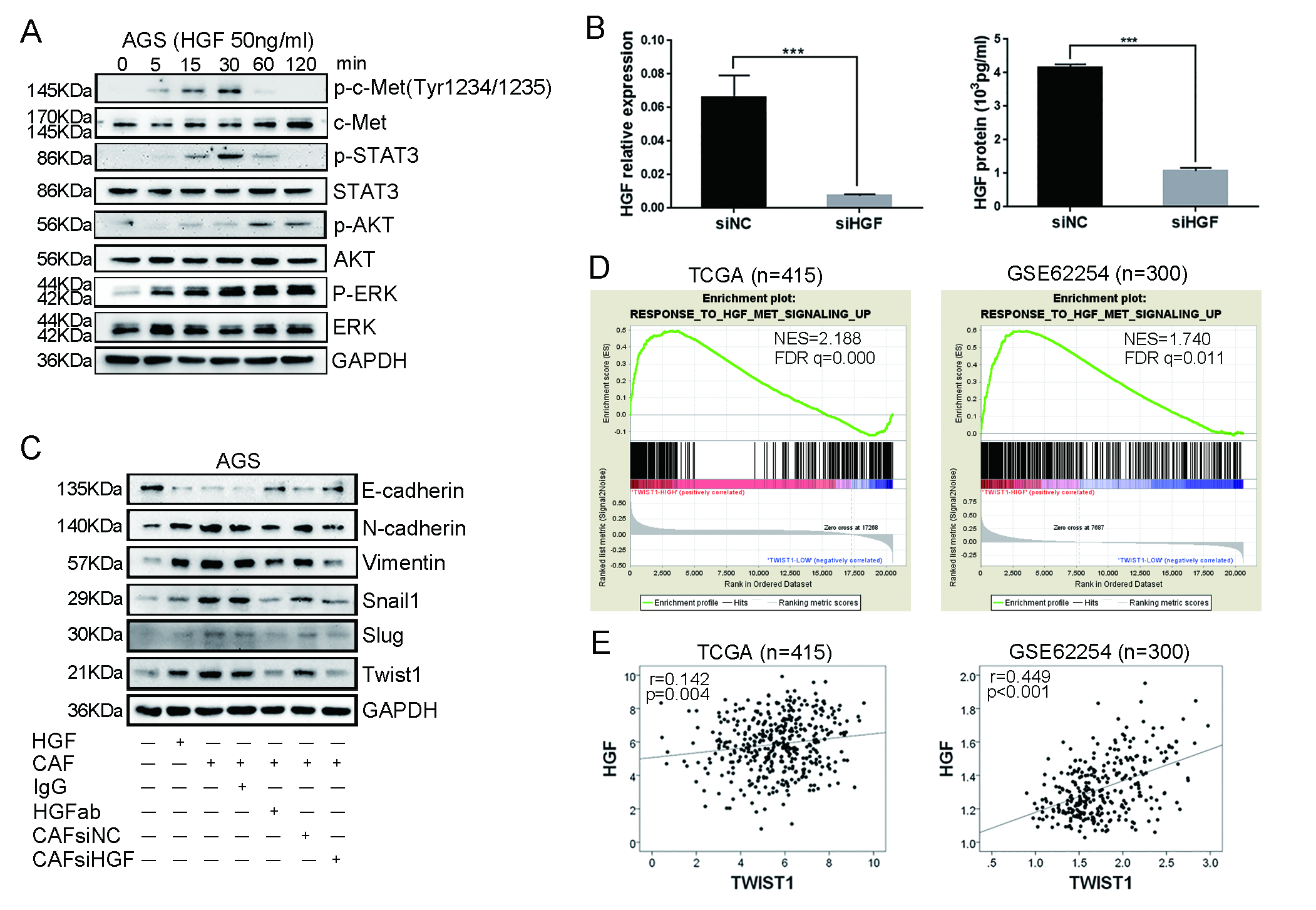

Supplement: Supplementary file 4 — Supplementary Figure 4 [file 41419_2018_922_MOESM4_ESM.tif]

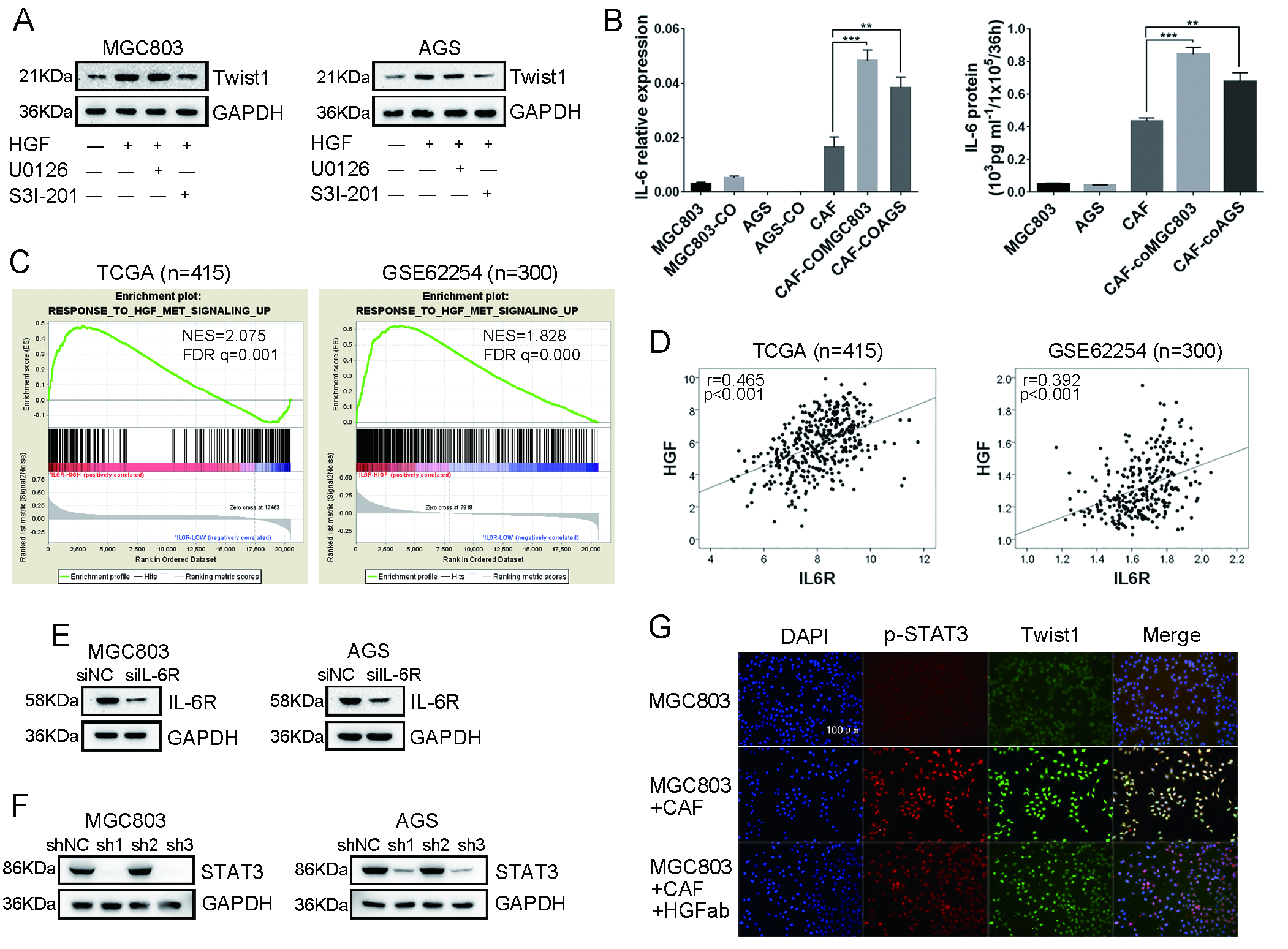

Supplement: Supplementary file 5 — Supplementary Figure 5 [file 41419_2018_922_MOESM5_ESM.tif]

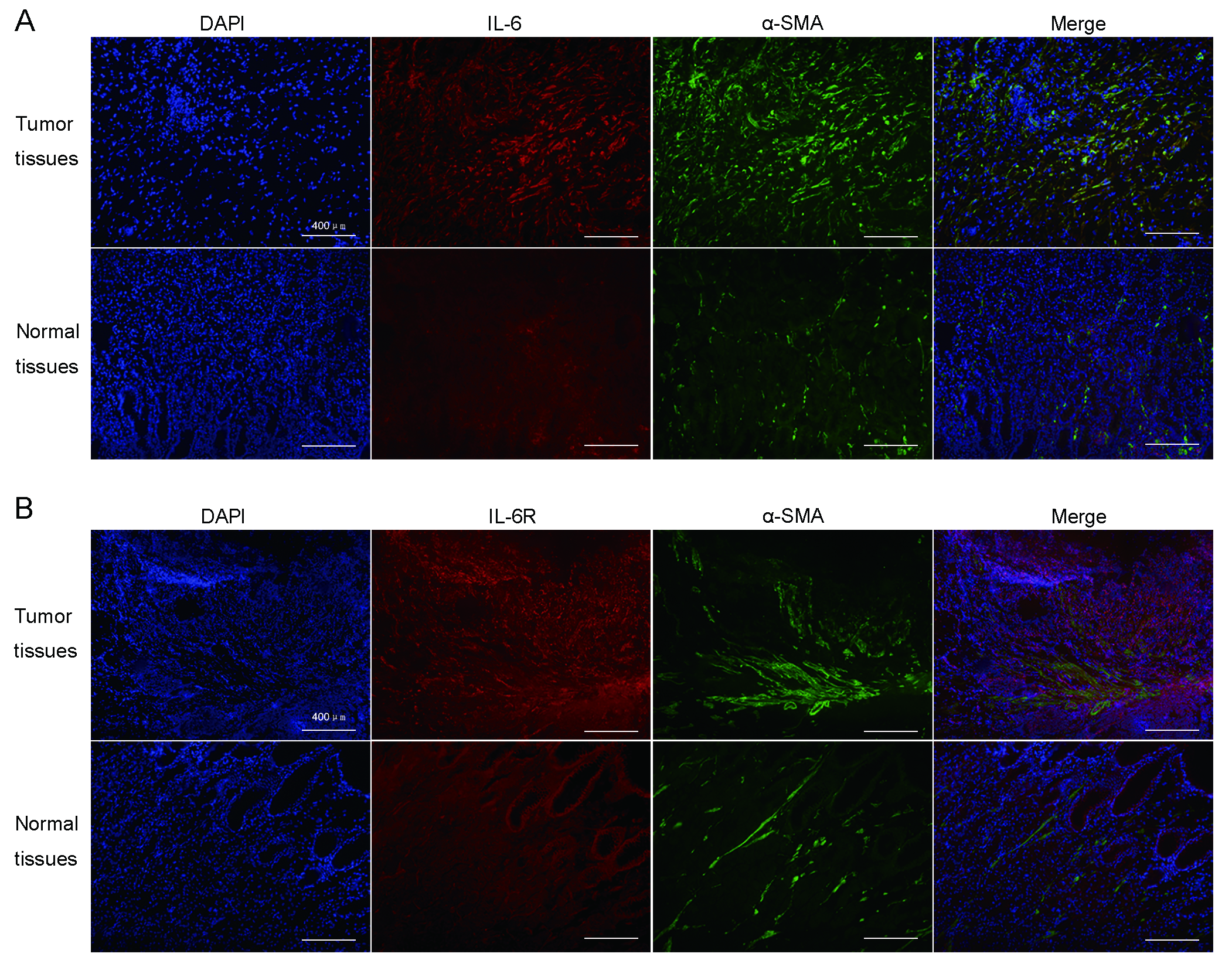

Supplement: Supplementary file 6 — Supplementary Figure 6 [file 41419_2018_922_MOESM6_ESM.tif]

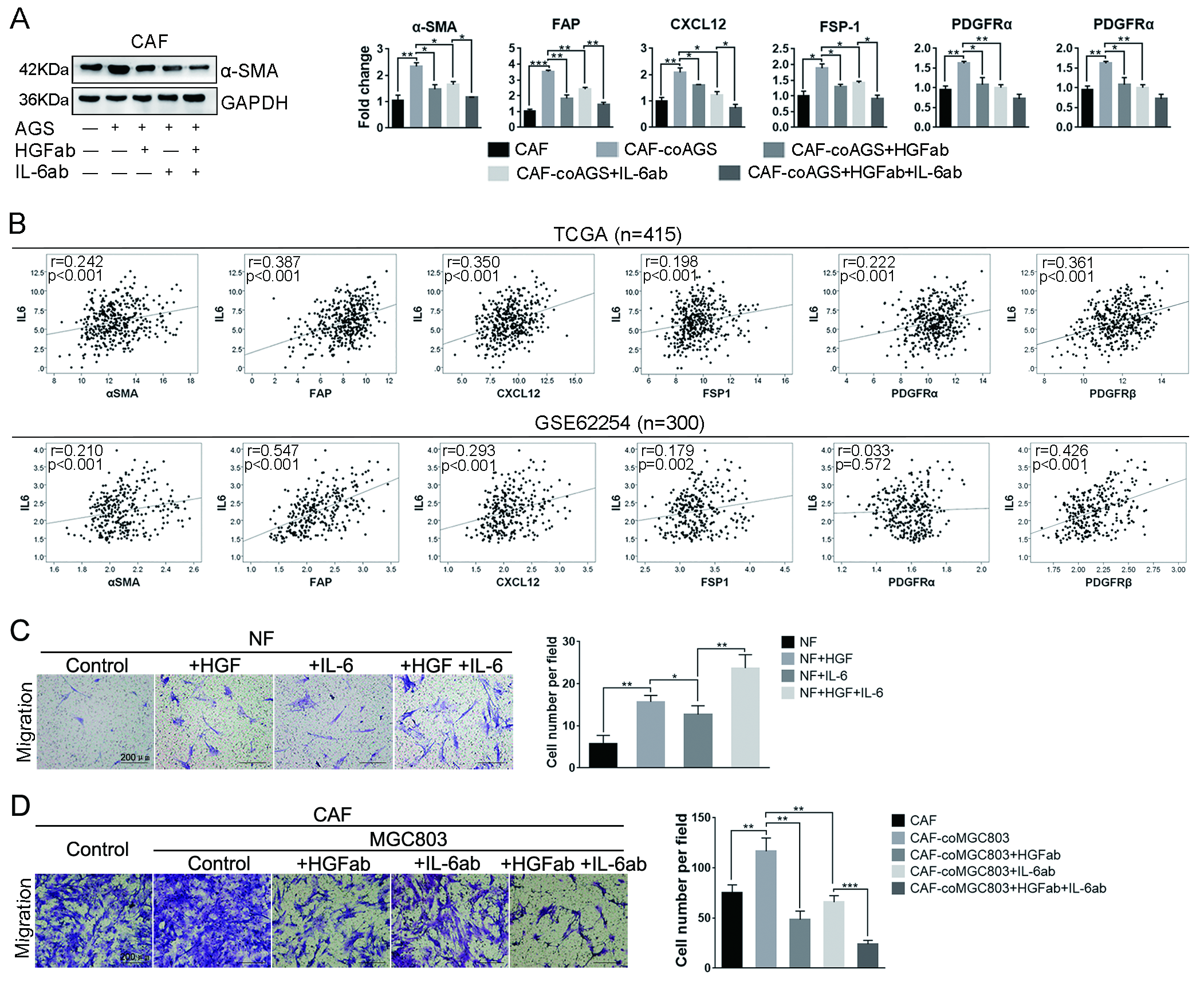

Supplement: Supplementary file 7 — Supplementary Figure 7 [file 41419_2018_922_MOESM7_ESM.tif]
