# Supplementary material for: HGF-mediated crosstalk between cancer-associated fibroblasts and MET-unamplified gastric cancer cells activates coordinated tumorigenesis and metastasis
Source: Cell Death Dis. 2018 Aug 29;9(9):867. doi: 10.1038/s41419-018-0922-1 (PMC6115420; doi:10.1038/s41419-018-0922-1)
Supplement: Supplementary file 8 — Supplementary Table 1 [file 41419_2018_922_MOESM8_ESM.doc]

**Table 1: Reagents for WB & IHC & FC & IF & ChIP & Stimulation & Inhibition**

| Name | Company and Product code | Concentration |
| --- | --- | --- |
| E-cadherin | CST, #3195 | 1:1000 (WB), 1:100(IHC) |
| N-cadherin | CST, #14215 | 1:1000 (WB) |
| Vimentin | CST, #5741 | 1:1000 (WB), 1:100 (IF), 1:100 (IHC) |
| Snail1 | CST, #3879 | 1:1000 (WB) |
| Slug | CST, #9585 | 1:1000 (WB) |
| c-Met | CST, #8198 | 1:2000 (WB), 1:200 (IF), 1:200 (FC) |
| p- c-Met | CST, #3077 | 1:2000 (WB), 1:200 (FC) |
| ERK1/2 | CST, #9102 | 1:1000 (WB) |
| p-ERK1/2 | CST, #9106 | 1:1000 (WB) |
| AKT | CST, #4691 | 1:1000 (WB) |
| p-AKT | CST, #4060 | 1:1000 (WB) |
| STAT3 | CST, #4904 | 1:2000(WB),1:50(ChIP),1:200 (IHC) |
| p-STAT3 | CST, #9145 | 1:2000 (WB), 1:100 (IF), 1:200 (IHC) |
| Pan-cytokeratin | CST, #4545 | 1:100 (FC) |
| Crizotinib | CST, #4401 | 0.1Μm (Inhibition) |
| U0126 | CST, #9903 | 20μM (Inhibition) |
| LY294002 | CST, #9901 | 50μM (Inhibition) |
| HGF | Abcam, ab83760 | 1:100 (IF), 1:100 (IHC) |
| IL-6 | Abcam, ab6672 | 1:100 (IF), 1:100 (IHC) |
| α-SMA | Abcam, ab5694 | 1:500 (WB), 1:100 (IF), 1:100 (IHC) |
| α-SMA | Abcam, ab7817 | 1:100 (IF) |
| Twist1 | Abcam, ab175430 | 1:1000 (WB), 1:100 (IF), 1:200(IHC) |
| S3I-201 | Abcam, ab141434 | 100μM (Inhibition) |
| Recombinant human HGF | Abcam, ab105061 | 50 ng/ml (Stimulation) |
| Recombinant human IL-6 | ABclonal, 10395-HNAE | 10 ng/ml (Stimulation) |
| Human HGF antibody | R&D, #24612 | 300 ng/ml (Inhibition) |
| Human IL-6 antibody | R&D, #1936 | 150 ng/ml (Inhibition) |
| IL-6R | SantaCruz, sc-373708 | 1:500 (WB), 1:100 (IF) |
| FAP | SantaCruz, sc-71094 | 1:100 (IF) |
| CD31 | SantaCruz, sc-65260 | 1:100 (FC) |
| CD45 | SantaCruz, sc-52386 | 1:100 (FC) |
| Ki67 | DAKO, MIB-1 | 1:50 (IHC) |
| AG490 | Medchem, HY-12000 | 10μM (Inhibition) |
| GAPDH | Proteintech, 60004-1-lg | 1:5000 (WB) |
