# Supplementary material for: HGF-mediated crosstalk between cancer-associated fibroblasts and MET-unamplified gastric cancer cells activates coordinated tumorigenesis and metastasis
Source: Cell Death Dis. 2018 Aug 29;9(9):867. doi: 10.1038/s41419-018-0922-1 (PMC6115420; doi:10.1038/s41419-018-0922-1)
Supplement: Supplementary file 9 — Supplementary Table 2 [file 41419_2018_922_MOESM9_ESM.doc]

| **For qRT-PCR** | | |
| --- | --- | --- |
| Name | Forward primer | Reverse primer |
| EGF | TGTCCACGCAATGTGTCTGAA | CATTATCGGGTGAGGAACAACC |
| VEGF | TTGCTGCTCTACCTCCAC | AATGCTTTCTCCGCTCTG |
| IGF-I | TGGACACGCTGCAGTTTGTGTGT | CACTCGTCCACAATACCACGGT |
| TNFA | CCTCTCTCTAATCAGCCCTCTG | GAGGACCTGGGAGTAGATGAG |
| bFGF | ATAAGCTTCAACTCCAGGCGACC | AGCATTCATCTGTTGTCCGTCTC |
| FGF4 | GTCGGTCAGGCGGTCAGT | CATGCAGCCGGGGTAGAG |
| FGF6 | CATAATGAAAACCAGTACAGTCTA | CGTATAACCTTCCTCGGCTATT |
| FGF9 | TCTACCTCGGCATGAATGAGAA | TGGAGGAGTACGTGTTGTACCA |
| GCSF | GCTGCTTGAGCCAACTCCATA | GAACGCGGTACGACACCTC |
| MCSF | AGACCTCGTGCCAAATTACATT | AGGTGTCTCATAGAAAGTTCGGA |
| GMCSF | TCCTGAACCTGAGTAGAGACAC | TGCTGCTTGTAGTGGCTGG |
| SCF | AATCCTCTCGTCAAAACTGAAGG | CCATCTCGCTTATCCAACAATGA |
| IL1A | TGGTAGTAGCAACCAACGGGA | ACTTTGATTGAGGGCGTCATTC |
| IL1B | AGCTACGAATCTCCGACCAC | CGTTATCCCATGTGTCGAAGAA |
| MCP1 | CAGCCAGATGCAATCAATGCC | TGGAATCCTGAACCCACTTCT |
| EOTAXIN1 | CCCCTTCAGCGACTAGAGAG | TCTTGGGGTCGGCACAGAT |
| CXCL9 | CCAGTAGTGAGAAAGGGTCGC | AGGGCTTGGGGCAAATTGTT |
| LEPTIN | TGCCTTCCAGAAACGTGATCC | CTCTGTGGAGTAGCCTGAAGC |
| ANGIOGENIN | CTGGGCGTTTTGTTGTTGGTC | GGTTTGGCATCATAGTGCTGG |
| HGF | GCTATCGGGGTAAAGACCTACA | CGTAGCGTACCTCTGGATTGC |
| IL6 | CGGTCCAGTTGCCTTCTCCC | GAGTGGCTGTCTGTGTGGGG |
| ACTA2 | CAGGGCTGTTTTCCCATCCAT | ACGTAGCTGTCTTTTTGTCCC |
| FAP | ACGGCTTATCACCTGATCGG | AATTGGACGAGGAAGCTCATTT |
| FSP1 | GATGAGCAACTTGGACAGCAA | CTGGGCTGCTTATCTGGGAAG |
| CXCL12 | ATGCCCATGCCGATTCTTCG | GCCGGGCTACAATCTGAAGG-3 |
| PDGFRA | AACCGTGTATAAGTCAGGGGA | GCATTGTGATGCCTTTGCCTT |
| GAPDH | GGACCTGACCTGCCGTCTAG | GTAGCCCAGGATGCCCTTGA |
| **For siRNA** | | |
| Name | Sense | Anti-sense |
| siNC | UUCUCCGAACGUGUCACGUTT | ACGUGACACGUUCGGAGAATT |
| HGF siRNA | CCGCUGGGAGUACUGUGCAAUUAAA | UUUAAUUGCACAGUACUCCCAGCGG |
| IL-6R siRNA | CGACUCUGGAAACUAUUCATT | UGAAUAGUUUCCAGAGUCGTG |
| **For semi-qRT-PCR of CHIP** | | |
| Name | Forward primer | Reverse primer |
| TWIST1 promoter | CAGTCTCCTCCGACCGCTT | GTGAGGAGGAGGGACTTTTCG |
