# Supplementary material for: HGF-mediated crosstalk between cancer-associated fibroblasts and MET-unamplified gastric cancer cells activates coordinated tumorigenesis and metastasis
Source: Cell Death Dis. 2018 Aug 29;9(9):867. doi: 10.1038/s41419-018-0922-1 (PMC6115420; doi:10.1038/s41419-018-0922-1)
Supplement: Supplementary file 10 — Supplementary Figure Legends [file 41419_2018_922_MOESM10_ESM.doc]

**Supplement Figure 1**

**HGF expression increases in GC tissues and is correlated with stromal fibroblasts.** (A) Expression of pan-cytokeratin, CD31 and CD45 in CAFs and NFs were measured by FC. Lines and areas were used to indicate protein expression: black dotted lines for isotype-control IgG; red solid lines for corresponding interest proteins. (B) HGF and α-SMA mRNA expression increased in GC tissues. (C) HGF expression was positively correlated with α-SMA expression on mRNA level. (*P < 0.05)

**Supplement Figure 2**

**c-Met expression increases in tumor tissues and MET amplification accounts for major part of gene alteration in GC.** (A) Representative images of celluar co-localization of c-Met (red) and α-SMA (green) by immunofluorescence stain with frozen tissue sections from GC and adjacent non-tumor tissues. Red color by Alexa Fluor 594 and green color by Alexa Fluor 488. Scale bars, 400 μm. (B) mRNA expression pattern across more than 21,000 samples profiled by Affymetrix U133plus2 platforms was analysed in Gene Expression across Normal and Tumour tissues (GENT). N, normal; C, cancer. (C) 20,981 tumour samples from TCGA was analysed for MET gene alteration in cBioportal for Cancer Genomic. Green, mutation; purple, fusion; red, amplification; blue, deep deletion; gray, multiple alterations. (D) Correlations of MET alteration with Overall Survival and Disease Free Survival were analysed in cBioportal Web resource online.

**Supplement Figure 3**

**Inhibition effects of c-Met TKI, crizotinib, on baseline and HGF-enhanced cell proliferation of GC cells, and toxic effects of crizotinib on GC cells and CAFs.**

**Supplement Figure 4**

**HGF/c-Met signaling induces EMT of MET-unamplified GC cells.** (A) Downstream signaling pathways activated by HGF. (B) The expression levels of HGF mRNA and protein in CAFS transfected with HGF siRNA were determined by qRT-PCR and ELISA, respectively. (C) HGF induced EMT of AGS cells. AGS cells were lysed after treatment with recombinant human HGF protein for two days or co-cultured with CAFs for two days. (D) GSEA of GC samples from TCGA and GSE62254 showed that HGF/c-Met signaling was positively correlated with twist1 expression. (E) Correlation analysis using GC samples from TCGA and GSE62254 showed that HGF expression was positively correlated of with twist1 expression. HGF (50ng/ml); HGFab (300ng/ml). (***P < 0.001)

**Supplement Figure 5**

**IL-6R/STAT3 signaling participates in HGF-induced twist1 expression.** (A) Twist1 expression in MET-unamplified GC cells were detected by western blotting. GC cells were pretreated with inhibitors for 6h, and the same concentration of these inhibitors were added into medium for two days. (B) IL-6 mRNA and protein expression were measured by qRT-PCR and ELISA, respectively. (C) GSEA results of GC samples from TCGA and GSE62254 showed that HGF/c-Met signaling was positively correlated with IL-6R expression. (D) Correlation analysis using GC samples from TCGA and GSE62254 showed that HGF expression was positively correlated of with IL-6R expression. (E, F) IL-6R and STAT3 expression in MGC803 and AGS cells transfected with IL-6R siRNA and STAT3 shRNA were detected by western blotting, respectively. (G) Immunofluorescence stain showed co-expression of p-STAT3 (red) and twist1 (green) in MGC803 cells. Red color by Alexa Fluor 594 and green color by Alexa Fluor 488. Scale bars, 100 μm. HGF (50ng/ml); U0126 (20μM); S3I-201 (100μM). (**P < 0.01; ***P < 0.001)

**Supplement Figure 6**

**IL-6 and IL-6R expression increase in GC tissues and IL-6 is mainly expression in stromal fibroblasts.** (A, B) Representative images of co-localization of IL-6 (red) and α-SMA (green), IL-6R (red) and α-SMA (green) by immunofluorescence stain with frozen tissue sections from GC and patient-matched adjacent non-tumor tissues, respectively. Red color by Alexa Fluor 594 and green color by Alexa Fluor 488. Scale bars, 400 μm.

**Supplement Figure 7**

**HGF and IL-6 enhanced the characteristics of CAFs.** (A) HGF and IL-6 increased CAFs markers expression in AGS cells. (B) Positive correlation of IL-6 and CAFs markers were analysed with samples from TCGA and GSE62254. (C) HGF and IL-6 promoted cell migration of NFs. Scale bars, 200 μm. (D) HGF and IL-6 neutralization inhibited cell migration of CAFs. Scale bars, 200 μm. HGF (50ng/ml); HGFab (300ng/ml); IL-6 (10ng/ml); IL-6ab (150ng/ml). (*P < 0.05;**P < 0.01; ***P < 0.001)
